# Production of infectious reporter murine norovirus by VP2 *trans*-complementation

**DOI:** 10.1128/jvi.01261-23

**Published:** 2024-01-16

**Authors:** Ryoka Ishiyama, Kazuhiro Yoshida, Kazuki Oikawa, Reiko Takai-Todaka, Akiko Kato, Kumiko Kanamori, Akira Nakanishi, Kei Haga, Kazuhiko Katayama

**Affiliations:** 1 Department of Infection Control and Immunology, Laboratory of Viral Infection, Ōmura Satoshi Memorial Institute & Graduate School of Infection Control Sciences, Kitasato University, Tokyo, Japan; 2 Department of Aging Intervention, National Center for Geriatrics and Gerontology, Laboratory of Gene Therapy, and Laboratory for Radiation Safety, Aichi, Japan; 3 Department of Biology-Oriented Science and Technology, Kindai University, Wakayama, Japan; University of Michigan Medical School, Ann Arbor, Michigan, USA

**Keywords:** murine norovirus, VP2, reporter virus

## Abstract

Human norovirus (HuNoV) causes gastroenteritis, a disease with no effective therapy or vaccine, and does not grow well in culture. Murine norovirus (MNV) easily replicates in cell cultures and small animals and has often been used as a model to elucidate the structural and functional characteristics of HuNoV. An MNV plasmid-based reverse genetics system was developed to produce the modified recombinant virus. In this study, we attempted to construct the recombinant virus by integrating a foreign gene into MNV ORF3, which encodes the minor structural protein VP2. Deletion of VP2 expression abolished infectious particles from MNV cDNA clones, and supplying exogenous VP2 to the cells rescued the infectivity of cDNA clones without VP2 expression. In addition, the coding sequence of C-terminal ORF3 was essential for cDNA clones compensated with VP2 to produce infectious particles. Furthermore, the recombinant virus with exogenous reporter genes in place of the dispensable region of ORF3 was propagated when VP2 was constitutively supplied. Our findings indicate that foreign genes can be transduced into the norovirus ORF3 region when VP2 is supplied and that successive propagation of modified recombinant norovirus could lead to the development of norovirus-based vaccines or therapeutics.

**IMPORTANCE:**

In this study, we revealed that some of the coding regions of ORF3 could be replaced by a foreign gene and infectious virus could be produced when VP2 was supplied. Propagation of this virus depended on VP2 being supplied *in trans*, indicating that this virus could infect only once. Our findings help to elucidate the functions of VP2 in the virus lifecycle and to develop other caliciviral vectors for recombinant attenuated live enteric virus vaccines or therapeutics tools.

## INTRODUCTION

Norovirus (NoV) is a positive-sense single-stranded RNA virus of the *Caliciviridae* family. Human norovirus (HuNoV) is the major causative agent of acute gastroenteritis, with more than one million cases worldwide each year (1). Although recent progress in *in vitro* HuNoV propagation with intestinal organoid-based systems provides hope for controlling and preventing HuNoV infections, no therapeutics or vaccines are yet available for this virus (2). However, much of the knowledge on noroviral infection was derived from studies using the murine norovirus (MNV). Its etiology is similar to that of HuNoV (3, 4), and most importantly, it can be efficiently propagated in tissue culture (5). Studies with MNV have provided a foundation for understanding the mechanistic details of the noroviral infection process (4, 6).

The noroviral genome encodes three open reading frames (ORFs). ORF1 encodes a nonstructural polyprotein that is self-cleaved by its protease subunit to generate six proteins: N-term (NS1/2), NTPase (NS3), 3A-like (NS4), VPg (NS5), protease (NS6), and RNA-dependent RNA polymerases (RdRp) (NS7). ORF2 and ORF3 encode the major capsid protein VP1 and the minor capsid protein VP2, respectively. The coding sequence of an additional ORF in the MNV genome, ORF4, overlaps with ORF2 encoding virulence factor 1 (VF1), but in a different reading frame. These sequences are involved in MNV pathogenesis (7). Three regions in the MNV RNA genome form functional secondary structures that are important for viral propagation: the region overlapping with the 5′ untranslated region (UTR) and ORF1 region, the region at proximal ORF2 start codon, and the region overlapping with the 3′ distal ORF3 region and 3′ UTR (8, 9).

The viral capsid, about 35nm in diameter, is composed of 180 VP1 proteins that enclose the VPg-linked RNA genome at the 5′ end. The number of VP2 molecules in the virion is as yet unclear (10–12). HuNoV VP2 is predicted to align with the inner surface of the VP1 shell and bind the VP1 dimer, which assumes that a virion can accommodate 90 VP2 proteins (11). However, VP2 detection by western blotting of either the virion or a VLP preparation revealed that only a few VP2 proteins were present in each particle (10, 12).

Noroviral VP2 is a highly basic RNA-binding protein (10, 11, 13). It is also highly phosphorylated upon overexpression in insect cells (13). HuNoV VP2 amino acid sequences are divergent (14), and their functional domains are poorly understood. Studies on VP2 in feline calicivirus (FCV), which belongs to the Vesivirus genus in *Caliciviridae*, are highly suggestive of a role for noroviral VP2 in viral infections. Structural studies using cryoelectron microscopy and tomographic analyses revealed that, upon binding of the capsid to the cellular receptor JAM1, the internal VP2s were aligned and extended outward to form portals (15). These might provide an exit for viral RNA and suggest that VP2 is critical in the delivery of viral RNA to host cells. A genetic approach to examine VP2’s role in FCV using a viral mutant expressing VP2 showed that VP2 was essential in viral infection, and the lack of functional ORF3 could be complemented by co-expressing VP2 during the infection process (16).

The plasmid-based reverse genetics system enabled genetic engineering of the noroviral genome (17). Yet, the genome regions that allow sequence modifications are not fully understood because of the presence of undiscovered functional sequence elements. Genetic modifications by random mutagenesis using transposon-mediated insertions revealed that the coding regions of NS4 and VP2 accept foreign sequence insertions (18). Thus, unlike other regions in the noroviral genome sequence, the VP2 coding region can accept insertion of short sequences, such as the FLAG tag; however, it is unclear if a foreign gene can be inserted into the VP2 coding region. In this study, we established a system to evaluate the RNA sequence region of ORF3, which is essential for the formation of the infectious particles while complementing the VP2 protein *in trans*. Our findings allowed us to generate MNV reporter viruses carrying either fluorescent or luciferase genes.

## RESULTS

### Minor capsid protein, VP2, is required for forming infectious particles

**Fig 1:**
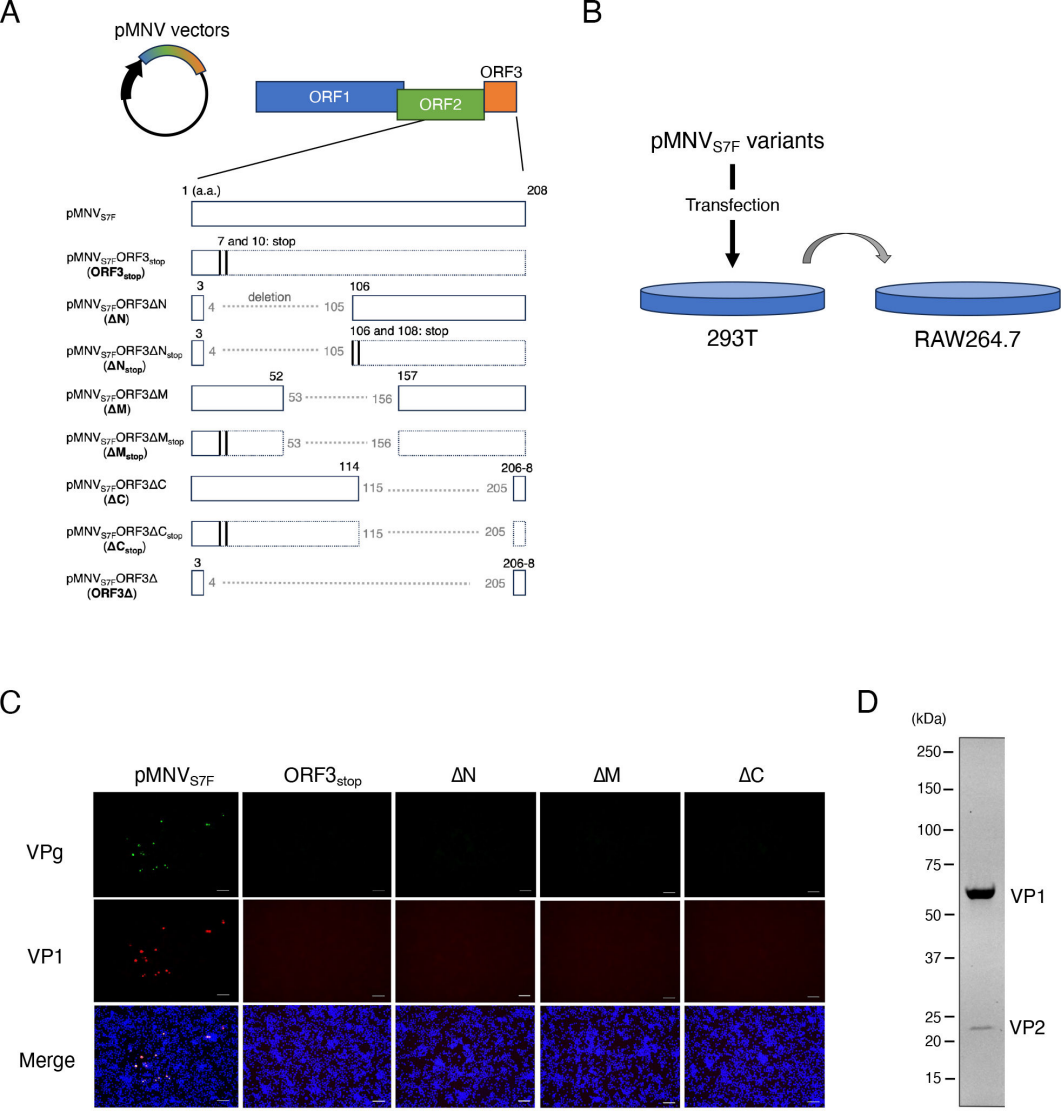
Deletion of VP2 protein prevented production of infectious MNV. (**A**) Schematic diagram showing mutations and deletions in the VP2 coding region of the pMNV_S7F_ vector. Numbers indicate the positions of the VP2 amino acid (a.a.) residues, and vertical lines with numbers indicate the positions of the insertion of the in-frame stop codon. Coding regions disrupted by stop codon are enclosed with dotted lines. (**B**) Schematic of the passages of MNV mutants. Each vector was transfected into 293T cells. After 48 hours of incubation, culture supernatants of transfected 293T cells were transferred to RAW264.7 cells. (**C**) Examination of infectious MNVs produced by each construct. Detection of infectious events in RAW264.7 cells by the infection of viral mutants defective in expressing VP2. Each indicated construct was transfected into 293T cells, and after 48 hours of incubation, each culture supernatant was transferred to RAW264.7 cells. After 24 hours of incubation, infectious events were detected by immunostaining for MNV VPg (green), VP1 (red), and nuclei (blue). The merged images are shown. Scale bars are 60 µm. This experiment was performed one time with three technical replicates. (**D**) Images were obtained using the ChemiDoc MP imager. Purified and concentrated MNV was loaded onto Mini-PROTEAN TGX Stain-Free Protein Gels (Bio-Rad) and electrophoresed. The band signal was obtained by detecting the fluorescence emitted by the reaction of the tryptophan residues of proteins with the excited trihalogenated compounds. This experiment was performed two times with one technical replicate.

Since ORF3 of MNV can accommodate the insertion of a FLAG tag (18), we examined VP2 function in virus infection to determine the essential portions of the VP2 region. This plasmid was also used to produce progeny MNV for reverse genetic engineering (17). For constructing the VP2-deleted virus, two stop codons were introduced at the 7th and 10th amino acid (pMNV_S7F_ORF3_stop_ (ORF3_stop_)) positions. For several truncated VP2-expressing mutants, in-frame deletions of the VP2 coding sequence were constructed from the 4th to the 105th (pMNV_S7F_ORF3ΔN (ΔN)), from the 53rd to the 156th (pMNV_S7F_ORF3ΔM (ΔM)), and from the 115th to the 205th (pMNV_S7F_ORF3ΔC (ΔC)) (Fig. 1A). These plasmid constructs were transfected into 293T cells, and 48 hours later, the culture supernatant was transferred to RAW264.7 cells (Fig. 1B). The original construct, pMNV_S7F_, produced progeny infectious MNV particles since viral proteins (VPg and VP1) were expressed in RAW264.7 cells. Constructs for VP2 protein deletion or each truncated-VP2 protein showed no viral protein expression in RAW264.7 cells (Fig. 1C).

Although HuNoV and MNV virus-like particles were produced without VP2 (19–21), infectious MNV particles required VP2 for infections and progeny production. Indeed, purified infectious MNV particles contained VP1 and VP2 (Fig. 1D); therefore, deletion or truncation of MNV VP2 disrupted the production of infectious particles. These findings indicated that deletions of approximately 100 amino acids in the ORF3 region prevented the formation of infectious particles.

### Functional complementation by supplying VP2 *in trans*

**Fig 2:**
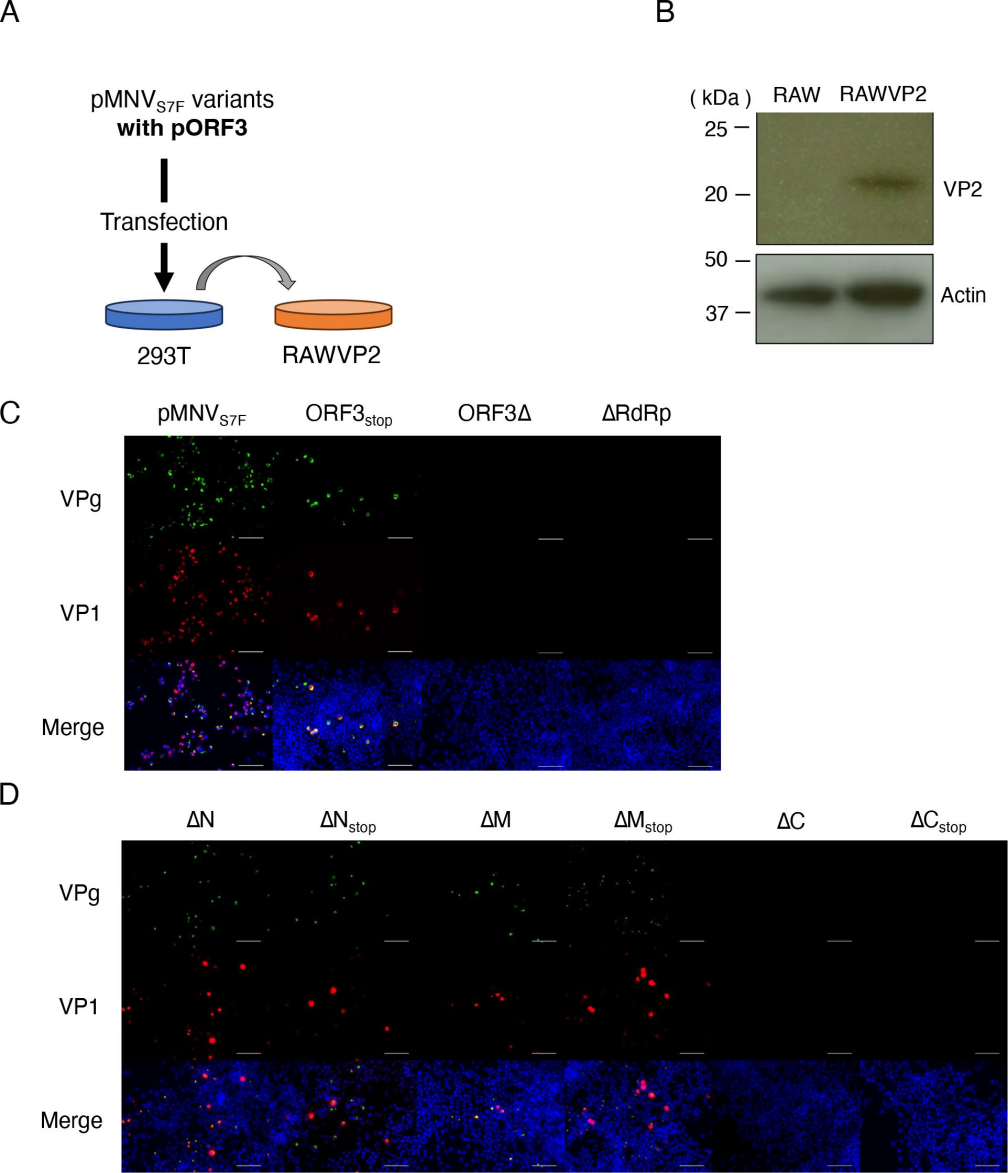
The sequence coding a.a. 115–205 of VP2 is essential for producing infectious MNV. (**A**) Schematic of the passages of the MNV mutants. Each vector was co-transfected with pORF3 to express VP2 into 293T cells. After 48 hours of incubation, culture supernatants of transfected 293T cells were transferred to RAWVP2 cells. (**B**) Western blotting to detect VP2 of RAWVP2 cells. The molecular mass is indicated at the left. Actin visualized with an antibody was used as a loading control. (**C and D**) Examination of infectious MNVs produced by pMNV_S7F_, ORF3_stop_, ORF3∆, and ∆RdRp constructs (**C**) and by ∆N, ∆N_stop_, ∆M, ∆M_stop_, ∆C, and ∆C_stop_ constructs (**D**). Detection of infectious events in RAWVP2 by the infection of viral mutants defective in expressing VP2. Each indicated construct was co-transfected with pORF3 into 293T cells, and after 48 hours of incubation, each culture supernatant was transferred to RAWVP2 cells. After 24 hours of incubation, infectious events were detected by immunostaining for MNV VPg (green), VP1 (red), and nuclei (blue). The merged images are shown. Scale bars are 60 µm. This experiment was performed one time with three technical replicates.

Since VP2 was required to produce infectious particles, exogenous VP2 protein was expressed in 293T cells and RAW264.7 cells, and a continuous supply of VP2 was provided throughout the post-transfection of each construct. The culture supernatant of 293T co-transfected with pMNV_S7F_ORF3_stop_ and pORF3 for VP2 expression was transferred to RAW264.7 cells expressing VP2 (RAWVP2) (Fig. 2A and B). After 48 hours of incubation, VPg and VP1 were expressed in RAWVP2 cells (Fig. 2C, “ORF3_stop_”), albeit a smaller number than in pMNV_S7F_ (Fig. 2C, “pMNV_S7F_”). This indicated a partial recovery of the infectious particles derived from ORF3_stop_ that lacked VP2 expression. By contrast, the construct lacking most of the ORF3 sequence (pMNV_S7F_ORF3Δ (ORF3Δ)) or lacking RdRp expression (pMNV_S7F_ΔRdRp (ΔRdRp)) could not be recovered by VP2 *trans* complementation (Fig. 2C, “ORF3Δ” and “ΔRdRp”).

Next, we determined the RNA coding sequences of ORF3 that were necessary for forming infectious particles under exogenous VP2-expressing conditions. In addition to the ΔN, ΔM, and ΔC constructs, pMNV_S7F_ΔN_stop_ (ΔN_stop_), pMNV_S7F_ΔM_stop_ (ΔM_stop_), and pMNV_S7F_ΔC_stop_ (ΔC_stop_) were designed by introducing two stop codons immediately after each start codon to eliminate the truncated VP2 proteins (Fig. 1A). Infectious events were observed in RAWVP2 cells that were incubated with the products derived from co-transfection of the ΔN, ΔM, ΔN_stop_ or ΔM_stop_ construct with pORF3 in 293T cells (Fig. 2D). Stop codon insertions in ΔN or ΔM did not significantly alter the efficiency of virus recovery, indicating that truncated VP2 protein from ΔN or ΔM did not impair the VP2 function supplied *in trans*. Meanwhile, exogenous VP2 expression could not rescue infectious particles from the ΔC construct (Fig. 2D). The failure of infectious events from RAWVP2 cells was not due to expression of C-terminal lacking VP2 protein from the ΔC construct because ΔC_stop_, which did not express truncated-VP2, also showed no infectious events in RAWVP2 cells. These findings indicated that the RNA sequence coding the 115th–205th amino acids of VP2 is essential for infectious events even with VP2 complementation.

### The ORF3 sequence coding amino acids 115 to 205 of VP2 included essential regions for infectious particle production

**Fig 3:**
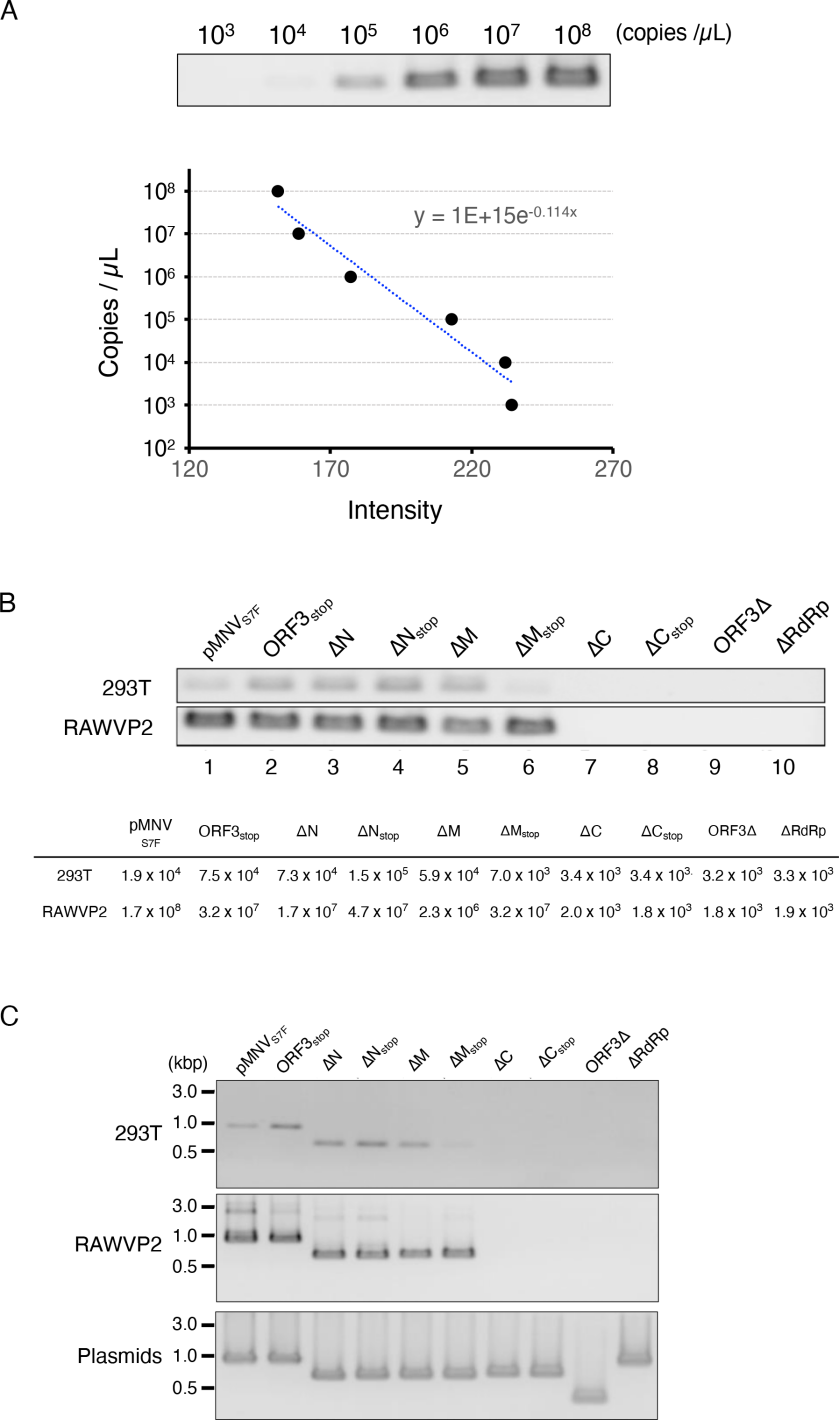
Detection of MNV RNA co-immunoprecipitated with anti-VP1 antibody from the cell culture medium. (**A**) Nested RT-PCR of standard RNA. Copies of MNV viral RNA synthesized *in vitro* were amplified in the ORF1/2 boundary region by the nested RT-PCR as the reference to the signal detected. An equation to determine the number of copies from the intensity of bands was obtained. This experiment was performed one time with one technical replicate. (**B**) Detection of viral RNA co-immunoprecipitation using anti-VP1 in the cell culture medium. Culture media of 293T cells transfected with each viral construct with pORF3 and that of RAWVP2 were immunoprecipitated with anti-VP1 antibody and detected for viral RNA by nested RT-PCR. Copy numbers were calculated from the intensity of bands. (**C**) ORF3 deletions were maintained during the propagation of the viral mutants harboring ORF3 mutations. The viral RNA was immunoprecipitated with an anti-VP1 antibody was amplified in the whole ORF3 region by nested RT-PCR. Top panel: Amplified products from the culture medium of 293T cells co-transfected with each viral construct and pORF3 (“293T”). Middle panel: Amplified products from the culture medium of the RAWVP2 (“RAWVP2”). Bottom panel: PCR amplification of the ORF3 region from the viral molecular clones harboring mutations in ORF3 (“Plasmid”). Each experiment was performed one time with one technical replicate.

It was unclear whether the reason for the loss of infectivity was the formation of non-infectious particles or failure of particle formation. If MNV genomic RNA was co-immunoprecipitated with VP1 protein, viral particles with genome RNA would be present in the culture supernatant. Viral particles in the culture medium were immunoprecipitated with an anti-VP1 antibody, the RNA was purified, and the RNA copies of the MNV genome were counted. Copy numbers of viral RNA were quantified by nested RT-PCR with a known copy number (Fig. 3A), and subsequently, the RNA copies were calculated from the intensity of the detected bands (Fig. 3B). Detection of co-immunoprecipitated viral RNA in the culture supernatants of transfected-293T cells by anti-VP1 antibody was consistent with results in Fig. 2D showing the infectious events in RAWVP2 cells (Fig. 3B). Co-immunoprecipitated viral RNA was more abundant in RAWVP2 culture medium than in 293T cells, meaning more particles appeared to be produced after passaging (Fig. 3B, lanes 1–6). Clones ΔC, ΔC_stop_, ORF3∆, and ∆RdRp yielded no detectable RNA by the immunoprecipitation with anti-VP1 antibody (Fig. 3B, lanes 7–10). In other words, they failed to form particles including genome RNA in culture supernatant even in the presence of VP2 supply *in trans*. These findings suggested that the deficiency was not due to the formation of non-infectious particles.

### Infectious events were not caused by revertants that reacquired functional VP2 coding region

The next step was to determine whether the observed infectious events were due to revertants, which frequently occur (16). Thus, the length of the truncated-VP2 coding sequence was confirmed by amplifying the entire ORF3 region of each viral mutant by RT-PCR. Cell-culture media, including the virus produced from the cells, either transfected with the mutant viral constructs and pORF3 (Fig. 3C, “293T”) or infected in the presence of VP2 (Fig. 3C, “RAWVP2”), were immunoprecipitated with anti-VP1 antibody. Viral RNA was purified from the immunoprecipitates; subsequently, the ORF3 region was amplified. In agreement with the previous results, ORF3_stop_, ΔN, ΔN_stop_, ΔM, and ΔM_stop_ appeared to generate viral particles carrying viral RNA, and passage in RAWVP2 yielded even more. By comparing these to the fragment size of the original plasmid construct (Fig. 3C, “Plasmid”), we found no obvious change in the ORF3 region of the mutants. This finding indicated that the introduced deletions were maintained during serial passage and no detectable revertants were generated during the experiments.

### Evaluation of VP2 complementation system using Huh7.5.1/CD300lf cells

To generate and propagate genetically modified viruses efficiently, the Huh7.5.1 cell-based MNV culture system was established for greater virus replication efficiency than RAW264.7 cells. Huh7.5.1 cells, a subline of the Huh7 hepatocarcinoma cell line, have a missense mutation in the RIG-I gene and are highly permissive to the JFH-1 strain of the hepatitis C virus (22). An MNV receptor molecule, mouse CD300lf, was transduced into Huh7.5.1, and highly efficient clonal cells were obtained after single-cell cloning (Fig. S1A). In agreement with published reports that used similar cells (23), Huh7.5.1/CD300lf (HuhCD300lf) cells were fully permissive to MNV and could produce more MNV than RAW264.7 cells (Fig. S1B). Moreover, the MNV VP2 gene was transduced into HuhCD300lf cells, and the cells susceptible to MNV and expressing VP2 (HuhCD300VP2) were obtained after single-cell cloning (Fig. S1C). Thus, we expected more viruses to be produced by our modified reverse genetics system in this study.

**Fig 4:**
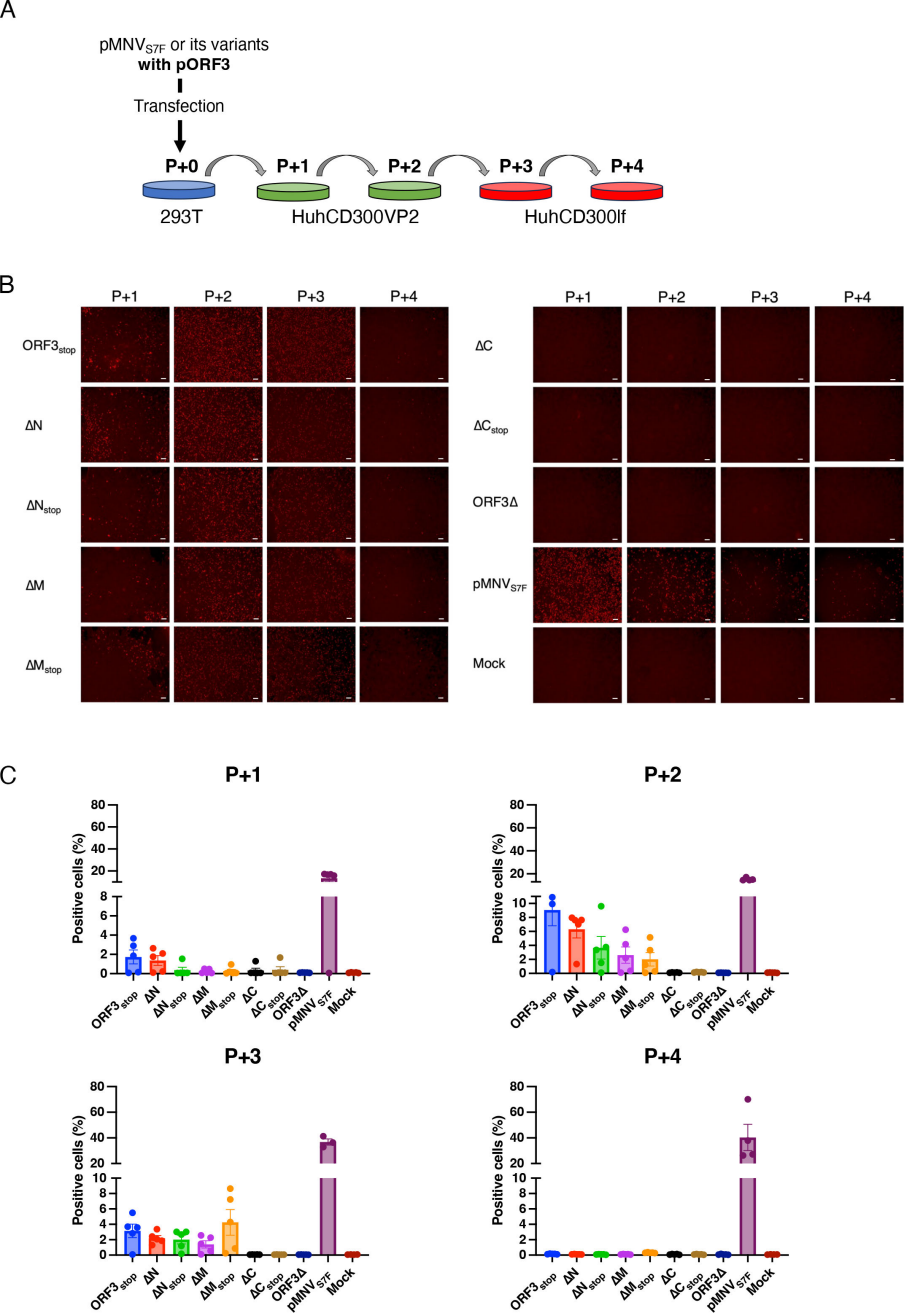
Huh7.5.1-based VP2 complementation system for propagating MNV VP2-defective mutants. (**A**) Schematic of the passages of the MNV mutants. Either MNV wild type or mutants defective in expressing VP2 generated by transfection to 293T cells with pORF3 were infected into HuhCD300VP2 cells (*P* + 1). The supernatant of P + 1 was passaged in HuhCD300VP2 cells (P + 2) and then twice in HuhCD300lf (P + 3 and P + 4). (**B**) Representative images of HuhCD300VP2 cells (P + 1 and P + 2) or HuhCD300lf cells (P + 3 and P + 4) inoculated with products from the cell culture medium of 293T cells after transfection with each construct plus pORF3. Cells in each passage were detected for infectious events by immunostaining of MNV NS1/2 protein (red). For each construct and passage, an image of each complete well was taken, and NS1/2 protein-positive cells were counted in five independent wells. Scale bars are 200 µm. (**C**) NS1/2 protein-positive HuhCD300VP2 cells (P + 1 and P + 2) or HuhCD300lf cells (P + 3 and P + 4) were counted from the whole image of each well using the built-in image processing software of the BZ-X 800. The percentage of infected cells was calculated by dividing the number of cells expressing the NS1/2 protein by the total number of Hoechst33342-positive cells. Each dot represents the percentage of positive cells in each image, and the bar indicates the mean value of the counted NS1/2-expressing cells. Error bars denote standard deviation (SD). This experiment was performed one time with five technical replicates. All images used for measurements are shown in Fig. S2.

Culture supernatants of 293T co-transfected each construct with pORF3 for VP2 expression were transferred to HuhCD300VP2 (Fig. 4A), and cells expressing the NS1/2 protein were counted. At the first transfer to HuhCD300VP2 cells (P + 1), NS1/2 protein expression was observed from ORF3_stop_, albeit a smaller number than in pMNV_S7F_ [Fig. 4B and C (P + 1)]. After another passage in the presence of VP2, the number of positive cells increased [Fig. 4B and C (P + 2)]. These findings indicated that partial recovery of the infectious particles derived from ORF3_stop_ was also observed in HuhCD300VP2 and that the virus was propagated. We also counted the cells expressing the NS1/2 protein to determine whether other mutant constructs produced infectious particles when VP2 was supplied. Infectious events were observed at the first transfer to HuhCD300VP2 cells that were incubated with the products derived from co-transfection of ΔN, ΔM, ΔN_stop_, or ΔM_stop_ construct with pORF3 in 293T cells [Fig. 4B and C (P + 1)]. Further passages to other HuhCD300VP2 cells increased the number of cells positive for viral protein [Fig. 4B and C (P + 2)]. However, the number of positive cells was lower when the cells were passaged to HuhCD300lf that was not supplied with VP2, and another passage yielded no positive cells [Fig. 4B and C (P + 3 and P + 4)], indicating that infectivity of this mutant virus depended on VP2 complementation. Meanwhile, VP2 complementation could not recover infectious particles provided from ΔC and ΔC_stop_ (Fig. 4B and C), which was the same result observed in RAWVP2 cells, and wild-type (WT) MNV derived from pMNV_S7F_ could keep their infectivity without VP2 complementation. The higher percentage of infected cells with WT would be due to a reduction of total cell number by significant cell death.

### Production of reporter virus using VP2 complementation system with Huh7.5.1/CD300lf cells

**Fig 5:**
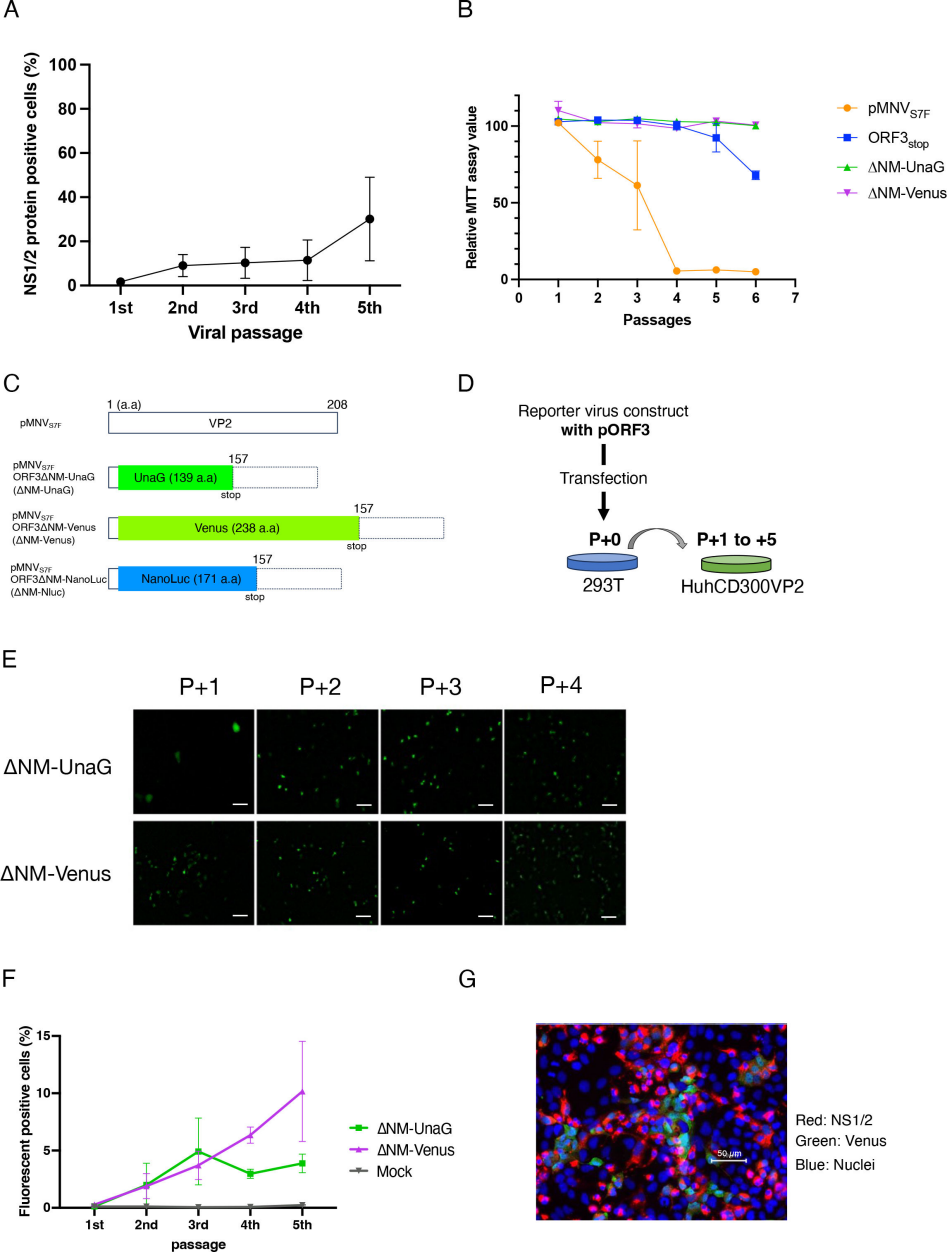
Propagation of MNV reporter virus in HuhCD300VP2 cells. (**A**) The mutant generated by co-transfection of ORF3_stop_ with pORF3 was passaged in HuhCD300VP2 cells. Cells in each passage were detected for infectious events by immunostaining of MNV NS1/2 protein. Five each passage, an image of each complete well was taken, and NS/1/2 protein-positive cells were counted in five independent wells. Circular dots indicate the mean value of the counted NS1/2-expressing cells. Error bars denote SD. This experiment was performed one time with five technical replicates. (**B**) Cell viability upon serial passages of MNV wild-type, VP2-defective mutant, and reporter viruses. HuhCD300VP2 was incubated with supernatant collected from each passage for 48 hours, and the extent of the formazan product converted from MTT by the cellular enzyme was examined by measuring the OD_490_. “Relative MTT assay value” indicates the proportion of the sample MTT assay value divided by that of the control cells that did not receive viruses at each time point. Each point indicates the mean value. Error bars denote SD. This experiment was performed one time with five technical replicates. (**C**) Schematic diagram of the reporter gene insertion into the VP2 coding region. Constructs used to generate the reporter viruses are listed at the left. Coding regions of reporter genes, UnaG and Venus, are highlighted in green, and NanoLuc is highlighted in blue. Numbers in the column indicate the positions of the VP2 amino acids. Boxes with dotted lines indicate untranslated VP2 coding regions due to the stop codon of the reporter gene. (**D**) Schematic of the passages of the MNV reporter mutants. MNV reporter mutants generated by transfection to VP2-expressing 293T cells were infected into HuhCD300VP2 cells (P + 1). The supernatant of P + 1 was repeatedly passaged in HuhCD300VP2 cells (P + 2 to P + 5). (**E**) Serial passages of the reporter viruses in HuhCD300VP2 cells. Reporter constructs (“∆NM-UnaG” and “∆NM-Venus”) were co-transfected with pORF3 to 293T cells; the culture media and the transfected cells were used as the sources of the virus. The reporter viruses ∆NM-UnaG and ∆NM-Venus were serially passaged in HuhCD300VP2 cells, and cells positive for the fluorescent signal were detected with an epifluorescent microscope. Scale bars are 150 µm. This experiment was performed one time with five technical replicates. (**F**) Fluorescent HuhCD300VP2 cells were counted from the five images randomly captured. Each point was the mean of the counted cells, and error bars denote SD. This experiment was performed one time with five technical replicates. (**G**) Cells were infected with the ∆NM-Venus reporter virus, fixed with 4% paraformaldehyde, and detected for NS1/2 protein (red), Venus fluorescence (green), and nuclei (blue).

Since the passage of the ORF3_stop_ virus in HuhCD300VP2 cells increased the number of cells expressing the NS1/2 protein, we repeated that virus passages among HuhCD300VP2 cells five times every 48 hours. After the 5th passage, the expression of NS1/2 protein was observed in about 40% of the cells (Fig. 5A) and decreased cell viability was observed in infected HuhCD300VP2 cells (Fig. 5B). Since decreased cell viability by the ORF3_stop_ virus was much less than by the WT, mutant virus production with exogenous VP2 was less efficient than WT, and at least, five or more passages were necessary to yield sufficient virus. Furthermore, propagated the ORF3_stop_ virus stably retained introduced the stop codon in ORF3 (Fig. S3A).

Our results consistently showed ∆N and ∆M viruses can propagate, but ∆C cannot, if VP2 was supplied *in trans* and led us to that the coding sequence of amino acids 4 to 156 of VP2 protein (ORF3^4th–156th^ sequence) could be replaced with a foreign gene. We replaced the ORF3^4th–156th^ sequence with a fluorescent protein- or luciferase-coding sequence and attempted to propagate modified viruses in HuhCD300VP2 cells. We prepared three constructs carrying the UnaG, Venus, and NanoLuc genes. UnaG is one of the smallest fluorescent proteins. Its fluorescence depends on the binding of bilirubin to fluorochromes (24). Venus is a variant of GFP that has been modified for better fluorescent properties (25). NanoLuc (Nluc) is a small luminescent enzyme modified for higher luminance than conventional luciferase (26). ∆NM-UnaG, ∆NM-Venus, and ∆NM-Nluc had a UnaG gene, Venus gene and Nluc gene inserted between the 4th and 156th residues of the VP2 coding region, respectively (Fig. 5C). Each construct was co-transfected with pORF3 into 293T cells to generate the reporter virus and, subsequently, the supernatant was passaged in HuhCD300VP2 cells (Fig. 5D). Upon reporter virus infection, we observed fluorescent signals in some HuhCD300VP2 cells that likely expressed UnaG or Venus (Fig. 5E). Although nearly 40% of cells were positive for the NS1/2 protein upon the 5th passage of VP2-defective mutants, fluorescence was detected in fewer than 10% cells after passages of the reporter viruses (Fig. 5A and F). Simultaneous detection of NS1/2 protein and Venus protein revealed that the cells visibly expressing Venus protein were underrepresented among the cells expressing NS1/2 protein (Fig. 5G).

According to these results, the inserted reporter gene may have been lacking during the serial passages. We determined whether the reporter viruses were stably retaining reporter genes during propagation using HuhCD300VP2. Genomic stability of the ∆NM-Venus and ∆NM-UnaG virus after five passages in HuhCD300VP2 was confirmed by next-generation sequencing (Fig. S3B and C). The ∆NM-Venus virus had no deletion, frameshifting or stop codon insertion in the ORF3 region, including the Venus gene. However, the ORF2 region had mutation T5691C (silent mutation) and T6598C (missense mutation; F515L) in 66% and 72% of the total reads, respectively. The ∆NM-UnaG virus had a deletion that removed the inserted UnaG gene in about 80% of the total reads. Therefore, we concluded that the small number of cells expressing UnaG protein was due to a lack of the UnaG gene. However, the Venus gene was maintained, and the reason that Venus-positive cells were underrepresented among the NS1/2 expressing cells was unclear.

To find out whether there were fewer cells expressing Venus protein than cells expressing NS1/2 protein, we attempted to count the number of cells expressing the NS1/2 and/or Venus protein by flow cytometry. HuhCD300lf cells, to restrict multiple infections, were infected with the ∆NM-Venus virus. After 24 hours post-infection (hpi), cells expressing the NS1/2 or Venus protein were measured by image analysis or flow cytometry (Fig. S4A and B). Little difference was observed in the ratios of the NS1/2 protein-expressing cells in the two methods. However, flow cytometry detected approximately 10 times more Venus-positive cells than image analysis (Fig. S4C). The Venus signal was probably lower than the fluorescent signal from NS1/2 protein staining, and some Venus-positive cells might have been missed by the BZ-X software from randomly captured images (Fig. S4A). Therefore, the ratio of Venus-positive cells shown in Fig. 5F is likely underestimated, and subsequent experiments for the ∆NM-Venus virus were evaluated using flow cytometry.

We next sought to confirm that the same levels of Venus-positive cells could be observed when infecting the ∆NM-Venus virus that had been further propagated. The ∆NM-Venus virus in different passages (P + 6, P + 7, and P + 8) was inoculated into HuhCD300VP2 cells, and the Venus-expressing cells were counted. Each passage of the ∆NM-Venus virus with the same number of RNA copies was infected into HuhCD300VP2 cells, and the population of Venus-expressing cells were 0.33%, 3.80%, and 6.19% at 24 hpi and 18.9%, 24.1%, and 25.3% at 48 hpi, respectively (Fig. S5). Therefore, since all passages yielded around 20% Venus-positive cells, the ∆NM-Venus virus was expected to retain the Venus gene in its genome even with further propagation.

### Characterization of reporter viruses

**Fig 6:**
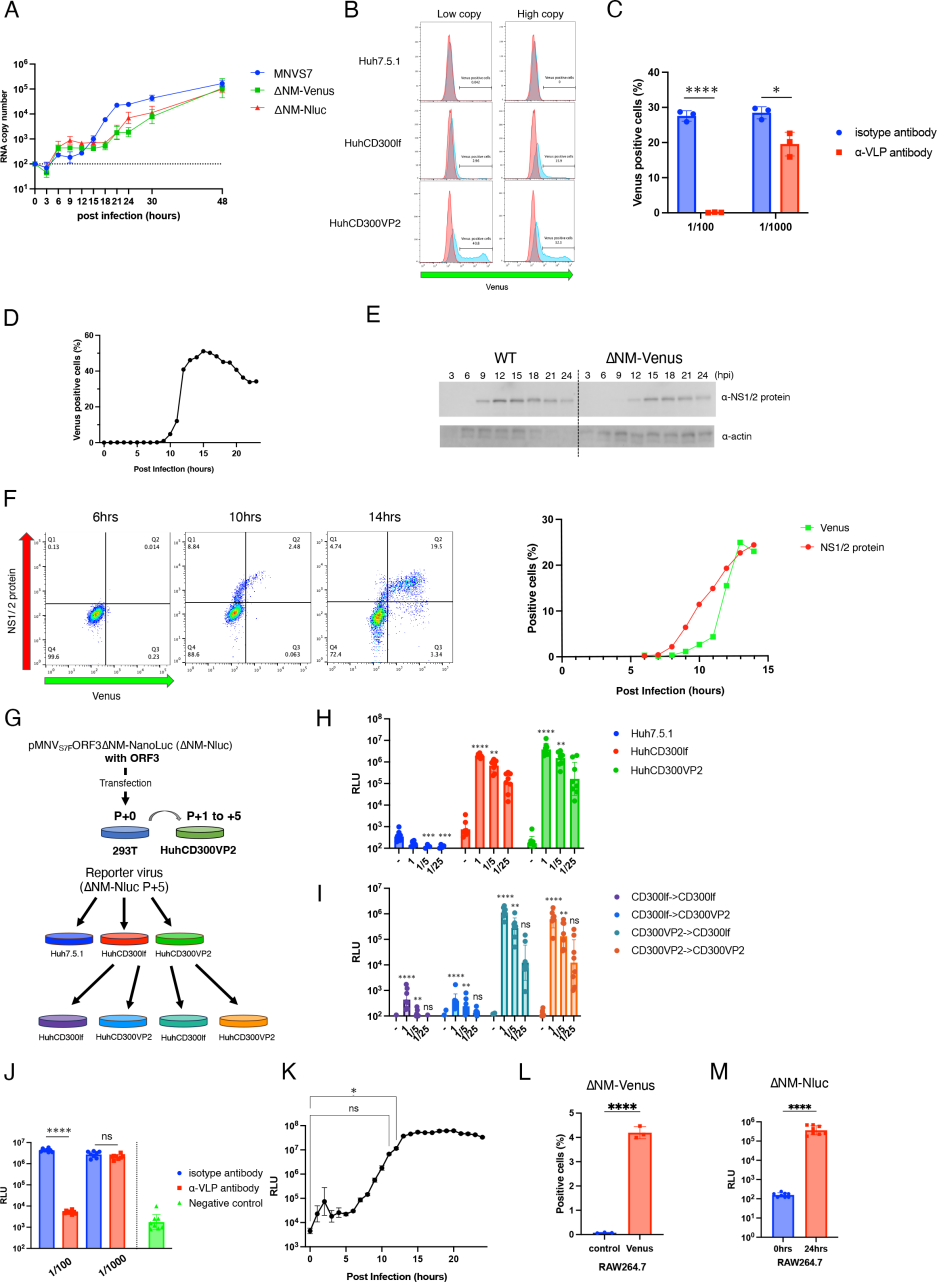
Characterization of the reporter viruses. (**A**) The reporter viruses and WT MNV (2 × 10^7^ copies) were infected into HuhCD300VP2 cells (2 × 10^4^ cells), and progeny production in the culture supernatant was quantified by quantitative PCR at the indicated times after infection. Each point was the geometric mean of the RNA copies, and error bars denote geometric SD. This experiment was performed one time with eight technical replicates. (**B**) The ∆NM-Venus virus (1 × 10^9^ or 1 × 10^8^ RNA copies) was infected with Huh7.5.1, HuhCD300lf, or HuhCD300VP2 cells (1 × 10^6^ cells). At 24 hpi, the population expressing Venus protein was measured by flow cytometry. This experiment was performed two times with one technical replicate. (**C**) The ∆NM-Venus virus (2 × 10^8^ RNA copies) was treated with diluted antisera (1/100 or 1/1,000) of rabbit immunized with MNV VLP or preimmunized and was inoculated to HuhCD300lf cells. Venus-expressing cells at 24 hpi were detected by flow cytometry. Significance was determined by Welch’s *t*-test (*P < 0.05, ****P < 0.0001). Each data bar represents the mean of three independent wells. Error bars denote SD. This experiment was performed one time with three technical replicates. (**D**) The ∆NM-Venus virus (1 × 10^9^ copies) was infected to HuhCD300lf cells (2 × 10^5^ cells), and infected cells were collected every hour after infection, and the population expressing Venus protein was measured by flow cytometry. This experiment was performed one time with one technical replicate. (**E**) The same copies of WT MNV or ∆NM-Venus virus (2 × 10^8^ copies) were infected into HuhCD300lf cells (2 × 10^5^ cells). Cell lysates were collected every 3 hours from 3 to 24 hpi. Actin was used as a loading control. (**F**) The ∆NM-Venus virus (1 × 10^8^ copies) was infected into HuhCD300lf cells (1 × 10^5^ cells). Cells were collected every hour at 6 to 14 hpi. Cells were fixed and permeabilized with methanol. After blocking, cells were treated with anti NS1/2 antibody and subsequently with Alexa 647 anti-rabbit IgG and analyzed by flow cytometry. Dot images at 6, 10, and 14 hpi are shown (images for every hour are available in Fig. S6), and the population transition expressing NS1/2 and Venus was graphed. This experiment was performed one time with one technical replicate. (**G**) Schematic showing the method of production and passages of the MNV reporter virus expressing NanoLuc luciferase (Nluc). The ∆NM-Nluc virus was produced by transfection into 293T cells with pMNV_S7F_-ORF3ΔNM-NanoLuc and pORF3. The supernatants of the transfected 293T cells (P + 0) were repeatedly passaged with HuhCD300VP2 cells (P + 1 to +5) for virus propagation. (**H**) After five propagations, each diluted virus solution [1 × 10^7^ (1), 2 × 10^6^ (1/5), 4 × 10^5^ (1/25) copies] was infected to each cell line (1 × 10^4^ cells), and luciferase activity was measured 24 hpi. (**I**) Furthermore, culture supernatants of the cells showing luciferase activity (HuhCD300lf and HuhCD300VP2 cells) were diluted (1, 1/5, or 1/25) and were passaged into HuhCD300lf and HuhCD300VP2 cells. The luciferase activity in each cell was assayed at 24 hpi using the Nano-Glo® Luciferase Assay System (Promega), and luminescence signal was detected by the luminometer. The vertical axis of the graph shows relative luminescence unit (RLU). Each data bar represents the geometric mean of eight independent wells. Error bars denote geometric SD. Each experiment was performed two times and a representative experiment is shown. Significance was determined by Kruskal-Wallis test (**P < 0.005, ***P < 0.0005, ****P < 0.0001, ns, not significant) (**J**) The ∆NM-Nluc virus (2 × 10^7^ RNA copies) was treated with diluted antisera (1/100 or 1/1,000) of rabbit immunized with MNV VLP or preimmune serum and inoculated into HuhCD300lf cells. Luciferase activities of infected cells were detected at 24 hpi. Significance was determined by Welch’s *t*-test (****P < 0.0001, ns, not significant). Each data bar represents the geometric mean of eight independent wells. Error bars denote geometric SD. This experiment was performed one time with eight technical replicates. (**K**) The ∆NM-Nluc virus (1 × 10^8^ copies) was infected into HuhCD300lf cells (2 × 10^4^ cells), infected cells were collected every hour after infection, and luciferase activity was measured. Significance was determined by the Kruskal-Wallis test (*, *P* < 0.05, ns, not significant). Each point was the geometric mean of RLU, and error bars denote geometric SD. This experiment was performed one time with eight technical replicates. (**L**) RAW264.7 cells (1 × 10^6^ cells) were infected with the ∆NM-Venus virus (1 × 10^9^ copies), and the population expressing Venus protein at 24 hpi was measured. Significance was determined by Welch’s *t*-test (****P < 0.0001). Each data bar represents the mean of three independent wells. Error bars denote SD. This experiment was performed one time with three technical replicates. (**M**) RAW264.7 cells (2 × 10^4^ cells) were infected with the ∆NM-NLuc virus (2 × 10^7^ copies), and the luciferase activity was measured at 24 hpi. Significance was determined by Welch’s *t*-test (****, *P* < 0.0001). Each data bar represents the geometric mean of eight independent wells. Error bars denote geometric SD. This experiment was performed one time with eight technical replicates.

To characterize the reporter viruses, first, progeny production was compared between WT MNV and reporter viruses when infecting HuhCD300VP2 cells. Lower and slower viral RNA release was observed in the reporter viruses than in the WT MNV (Fig. 6A). Next, to determine whether the ∆NM-Venus virus retained the MNV infectious properties, the ∆NM-Venus virus was inoculated into Huh7.5.1, HuhCD300lf or HuhCD300VP2, and the Venus-expressing cells were counted. In HuhCD300lf or HuhCD300VP2 cells, the ∆NM-Venus virus-induced fluorescence in a viral dose-dependent manner but there was no signal in the Huh7.5.1 cells (Fig. 6B). Moreover, antisera from rabbits immunized with MNV VLP inhibited the ∆NM-Venus virus infection and subsequent Venus gene expression in HuhCD300lf (Fig. 6C). In addition, the ∆NM-Venus virus was infected with HuhCD300lf and Venus-positive cells were counted every hour after infection. Venus protein could be detected at 10 hpi (Fig. 6D). These results suggest that the ∆NM-Venus virus has the infectious machinery using CD300lf, and Venus protein was detected in single-cycle replication because fluorescence signal was seen in HuhCD300lf cells. Recently, non-specific uptake of MNV RNA *in vivo* (27) was reported; however, uptake of free viral RNA that would be present in the culture supernatants did not occur in our system since Huh7.5.1 cell showed no fluorescence by the ∆NM-Venus virus inoculation. Since it was unclear that the ∆NM-Venus virus behaved like WT MNV after viral entry, we observed the kinetics of viral protein and reporter gene expression in the early stages of viral infection in HuhCD300lf cells. Equal RNA copies of WTMNV or ∆NM-Venus virus were infected to HuhCD300lf cells, and NS1/2 protein was detected by 9 or 12 hours, respectively (Fig. 6E). Moreover, NS1/2 protein- or Venus protein-expressing cells were also detected from 8 or 10 hpi, respectively, using the flow cytometry (Fig. 6F; Fig. S6). These findings indicated that the ∆NM-Venus virus replication efficiency seemed to be slower than that of WT MNV.

Next, we confirmed whether the ∆NM-Nluc virus also retained the MNV properties. First, the ∆NM-Nluc construct was co-transfected with pORF3 into 293T cells, the supernatant was repeated passaging five times with HuhCD300VP2 cells for viral propagation (Fig. 6G). Next, the genomic stability of the ∆NM-Nluc virus was determined by next-generation sequencing (Fig. S3D). At least 50% of the ∆NM-Nluc virus had one to five adenines inserted into the Nluc gene. Moreover, the ORF2 region had mutations G5119A (missense mutation; V22I) and C5821A (missense mutation; P256T) in 76% and 96% of the total reads, respectively. The C5821A mutation was already included in the plasmid construct. Even if at least 50% of the ∆NM-Nluc virus had one to five adenines inserted into the Nluc gene, the virus would be sufficient to investigate the properties of MNV. The culture supernatants after five passages were diluted to three levels (×1, ×5, or ×25) were inoculated into Huh7.5.1, HuhCD300lf, or HuhCD300VP2 cells, and the levels of luciferase activity were evaluated at 24 hpi. Luciferase activity increased after the ∆NM-Nluc virus infection in a dose-dependent manner in HuhCD300lf and HuhCD300VP2 but not in Huh7.5.1. cells (Fig. 6H), indicating that the ∆NM-Nluc virus infects cells with CD300lf-dependent machinery. Furthermore, each culture supernatant from cells showing obvious luciferase activity was inoculated into HuhCD300lf or HuhCD300VP2 cells again. Supernatants from HuhCD300VP2 cells showed increasing luciferase activity in both cell lines but neither showed luciferase activity when that from HuhCD300lf cells was inoculated (Fig. 6I). Moreover, antisera from rabbits immunized with MNV VLP inhibited the ∆NM-Nluc virus infection and subsequent Nluc gene expression in HuhCD300lf cells (Fig. 6J). In addition, we collected the cells every hour after infection to analyze the luciferase activity and found that luciferase activity significantly increased at 12 hpi when the ∆NM-Nluc virus was infected with HuhCD300lf cells (Fig. 6K). These findings suggested that the ∆NM-Nluc virus also infects through CD300lf as a receptor, the propagation of this virus requires exogenous VP2 expression, and luciferase activity can be detected in single-cycle replication in the whole cell population.

Finally, we determined whether the reporter viruses could be detected in natural hosts, RAW264.7 cells. When infected with the ∆NM-Venus virus, the population of Venus-expressing RAW264.7 cells were 4.20% ± 0.25 % at 24 hpi (Fig. 6L) and were less than that of Venus-expressing HuhCD300lf cells. Besides, when infecting the ∆NM-Nluc virus, infected RAW264.7 cells showed about 10 times less luciferase activity than HuhCD300lf cells (Fig. 6M). Although the reporter protein expressed in RAW264.7 was less than that in HuhCD300lf cells, the reporter virus could infect and the reporter protein could be detected in RAW264.7 cells.

## DISCUSSION

**Fig 7:**
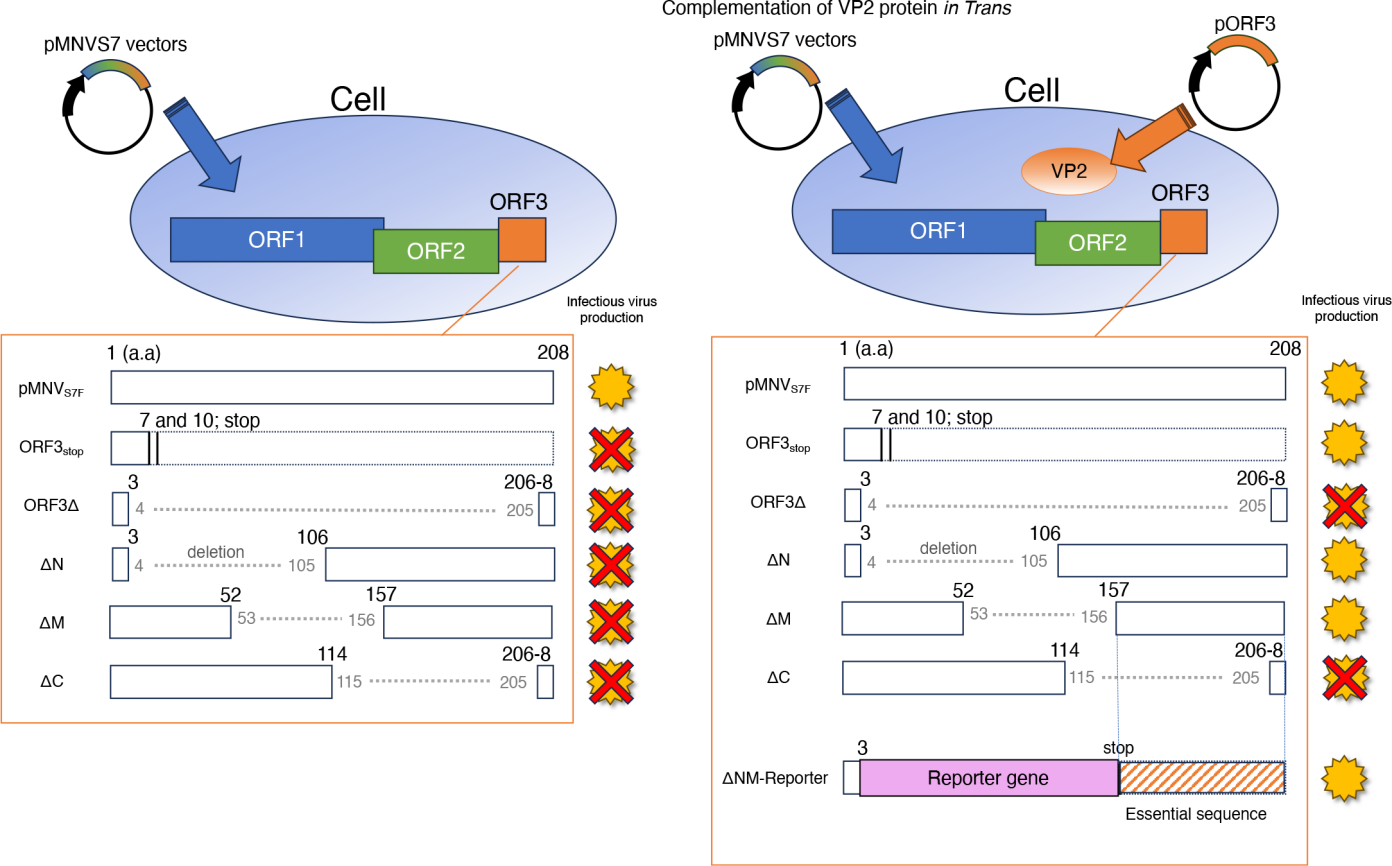
Summary figure of this study. The need for VP2 protein and ORF3 sequence in MNV production was evaluated separately. First, VP2 protein was essential for MNV production. The ORF3_stop_ mutant could be propagated but not the ORF3∆ mutant under VP2 suppling condition. Second, the ORF3^157th–208th^ sequence included essential regions for virus production, while the ORF3^4th-156th^ sequence was dispensable if VP2 protein was supplied *in trans*. The ORF3^4th–156th^ region could be replaced with a foreign gene and the replaced virus (the reporter virus) could be propagated under VP2 suppling conditions.

We used a genetic approach to examine the role of the VP2 protein in MNV infection. The needs for the VP2 protein and the RNA sequence in ORF3 for a productive MNV infection were analyzed separately (Fig. 7). First, a molecular clone of viral mutants was generated that lacked VP2 expression due to insertion of two stop codons immediately after first methionine of ORF3 (ORF3_stop_) (Fig. 1). No infections were noted after transferring culture supernatant of 293T cells transfected with the RGS construct not expressing VP2 protein. However, supplying VP2 protein exogenously in 293T cells and, subsequently, MNV permissive cells restored infectious viral production from ORF3_stop_ (Fig. 2 and 4), while whole ORF3 deletion (ORF3∆) was not restored, suggesting the presence of the essential ORF3 sequence region. Second, several molecular clones with a deletion of the ORF3 RNA sequence were transduced into 293T cells expressing VP2, and we determined that the coding sequence of amino acids 115–205 of the VP2 protein (ORF3^115th-205th^ sequence) was included essential region for infectious virus production because of failure of ∆C virus production. The culture supernatant of cells transduced with the ∆C construct did not include the viral RNA immunoprecipitated with anti-VP1 antibody, indicating that the formation of the viral particle containing viral RNA requires the ORF3^115th-205th^ sequence. Moreover, the apparent propagation of the mutant was not due to revertants: viral propagation was not observed when VP2 was not supplied *in trans,* and the nucleotide alterations were maintained during the passage of the mutant viruses (Fig. 3C and 4C; Fig. S3A). Meanwhile, ∆N and ∆M virus could be produced under VP2 complementation *in trans*, leading us to the ORF3^4th-156th^ sequence was dispensable and could be replaced with a foreign gene if VP2 was supplied. With HuhCD300VP2 cells, viral variants with genes encoding fluorescent or luciferase proteins were propagated and transduced to express the reporter genes in target cells. Thus, our study revealed some roles of VP2 in the viral lifecycle by exploiting the VP2 coding region and showed its potential as a noroviral reporter vector.

Approximately two-thirds of the proximal region of the VP2 coding region were not essential for viral propagation as long as VP2 was supplied *in trans*. Deletion of the ORF3^115th-205th^ sequence was detrimental to the viral lifecycle, even in the presence of VP2 protein. These results are consistent with previous findings in FCV, where VP2 supplied *in trans* could not rescue the deletion of the C-terminal VP2 coding region (16). In MNV, the ORF3^115th–205th^ sequence overlaps with a region predicted to form three stem-loop RNA secondary structures (8). The first stem loop, SL1, overlaps with the coding region of VP2 amino acid 166 to the end and was deleted in ΔC. Supplying VP2 *in trans* did not rescue the infection process of ΔC or ΔC_stop_, providing experimental evidence that the region encompassing the SL1-forming sequence is critical for the viral lifecycle. This had not been determined in previous studies (8, 9, 28).

A structural study of FCV entry suggested a role for MNV VP2 in the infection process. Upon FCV recognition by the cellular receptor JAM1, VP1 undergoes a conformational shift to expose the internal VP2, which self-assembles and forms a portal to release viral RNA from the viral capsid into the host cell (15). Note that the VP2s of FCV and MNV differ considerably in size and sequence. It is yet to be determined whether the VP2 of MNV has a role in viral entry similar to that in FCV. Alternatively, the lack of VP2 could compromise the structural integrity of the viral particles because VP2 contributes to the stability of the capsid structure (29). Assembly of the capsid is governed by VP1 and is carried out without VP2 and the viral RNA genome (19, 20). However, VP1 and VP2 directly interact in Norwalk virus (11) and FCV (15, 30), and in this study, purified MNV retained VP2 protein within virus particles (Fig. 1D). When VP2 was deleted in FCV, genomic RNA replication was not impaired in CRFK cells, but infectious progeny was not produced (16), indicating that FCV VP2 was involved in successful capsid assembly. Therefore, VP2 of MNV also may be involved in the efficient assembly or RNA incorporation into viral particles.

HuhCD300VP2 cells performed better in the propagation of mutant viruses. Serial passaging of ORF3_stop_ with HuhCD300VP2 cells showed viral antigen expression in approximately 40% of cells and evident cytopathic effects (Fig. 5A and B). Revertants were not observed in propagated ORF3_stop_ virus (Fig. S3A). Based on this success, the reporter viruses, which replaced the ORF3^4th–156th^ sequence with the reporter gene, were propagated in HuhCD300VP2 cells (Fig. 5C and D). The genomic stability of each propagated reporter virus was confirmed by whole genomic sequencing. In the ∆NM-UnaG virus, the UnaG gene was entirely deleted but the ∆NM-Venus virus had no mutation in the Venus gene. Although half of the population of the ∆NM-Nluc virus had one to five adenines inserted, the ∆NM-Nluc virus infection showed remarkable luciferase activity in the cell. The stability of the inserted gene may depend on the length of the inserted gene. Therefore, when generating the reporter virus in this system, the stability of the inserted gene in the viral genome needs to be confirmed after propagation. There were several mutations observed in the ORF2 region of the reporter virus. Although the replication speed of the reporter viruses seemed to be slower than the WT virus, these mutations did not change their MNV properties: they used CD300lf as a receptor and MNV antisera inhibited reporter virus infection. These results indicate that a few mutations accumulate during propagation but these recombinant viruses infect and express a detectable fluorescent protein or luciferase activity in the cell and have the potential to serve as a reporter virus.

Finally, the ∆NM-Venus virus and ∆NM-Nluc virus were infected with RAW264.7 cells that were derived from the natural host, the mouse. Both viruses showed detectable reporter gene expression after infection in RAW264.7 cells, albeit about 10-fold less in HuhCD300lf cells, and these findings indicate that the reporter virus could work *in vivo* and be used to study various aspects of MNV. Moreover, this system could also benefit the generation of HuNoV reporter vectors, which would be valuable for gaining insights into the virology of HuNoV. Furthermore, if our findings apply to other calicivirus, such as human norovirus or sapovirus that infect humans, they could lead to the development of an attenuated live viral vaccine that could be amplified using VP2-expressing cell lines and be expected to infect only once *in vivo* where VP2 is not supplied.

## MATERIALS AND METHODS

### Cell lines

The cell line, 293GP, from Riken Cell Bank, was maintained in Dulbecco’s Modified Eagle Medium (DMEM; Wako), supplemented with 10% fetal bovine serum (FBS) and MEM non-essential amino acid solution (Thermo Fisher Scientific). 293T (ATCC), RAW264.7 (ATCC), and Huh7.5.1 (a gift from Dr. Francis V. Chisari) cells were maintained in DMEM supplemented with 10% FBS.

### Construction of plasmids

The eight kinds of MNV VP2 coding region (ORF3)-modified plasmids that we used for the functional analysis of VP2 were constructed from pMNV_S7F_ (17), which is the murine norovirus S7 strain’s (Genbank accession number: AB435515) infectious clone plasmid, and it was used as the backbone plasmid. To supply VP2 protein *in trans* in the cells, pORF3 was also constructed from pMNV_S7F_ by deleting ORF1 and most of the ORF2 coding region.

VP2 mutations or deletions were introduced into the MNV cDNA of pMNV_S7F_. The 7th (GGA) and 10th (GGA) codons of ORF3 were changed to stop codons (TGA) to generate pMNV_S7F_ORF3_stop_ (ORF3_stop_). pMNV_S7F_ORF3ΔN (ΔN), pMNV_S7F_ORF3ΔM (ΔM), pMNV_S7F_ORF3ΔC (ΔC), and pMNV_S7F_ORF3Δ (ORF3Δ) were constructed by deleting ORF3 regions encoding amino acids 4–105, 53–156, 115–205, and 4–205, respectively. The constructs pMNV_S7F_ORF3ΔN_stop_ (ΔN_stop_), pMNV_S7F_ORF3ΔM_stop_ (ΔM_stop_), and pMNV_S7F_ORF3ΔC_stop_ (ΔC_stop_) were made from ΔN, ΔM, and ΔC, respectively, by inserting two stop codons (TGA) in place of the 4th (106th) and 6th (108th) codons for pMNV_S7F_ORF3ΔN_stop_ and 7th and 10th codons for the remaining two constructs.

The pMNV_S7F_ORF3ΔNM-UnaG (∆NM-UnaG), pMNV_S7F_ORF3ΔNM-Venus (∆NM-Venus), and pMNV_S7F_ORF3ΔM-NanoLuc (∆NM-Nluc) were made by replacing the ORF3 region encoding amino acids 5–156 with an in-frame insertion downstream of 4th VP2 codon sequence of UnaG, Venus, and NanoLuc coding regions with their stop codons, respectively.

MSCV-VP2-IRES-GFP, a mouse stem cell virus (MSCV) vector expressing VP2, was constructed from MSCV IRES GFP vector (Addgene #20672) by inserting the MNV VP2 coding region proximal to the internal ribosome entry site (IRES). pLVSIN-MNV VP2-IRES-puro was constructed to create a lentiviral vector expressing VP2 from pLNSIN-IRES-puromycin (31) by inserting the MNV ORF3 region.

The sequences of all the DNA constructs were confirmed by dideoxynucleotide sequencing (SeqStudio Genetic Analyzer, ABI) and next-generation sequencing (iSeq, Illumina).

### Production of RAWVP2, Huh7.5.1/CD300lf, and HuhCD300VP2 cells

RAWVP2 cells were generated by transducing the MSCV vector expressing MNV VP2 into RAW264.7 cells. The MSCV vector was produced by transfecting MSCV-VP2 IRESeGFP and VSV-G into 293GP cells using the Lipofectamine 3000 reagent (Thermo Fisher Scientific). At 48 hours after the transfection, the supernatant of 293GP containing the retroviral vector was collected and added to RAW264.7 cells, followed by incubation for 48 hours to generate RAW264.7VP2, herein RAWVP2.

Huh7.5.1/CD300lf (HuhCD300lf) cells were obtained from the Huh7.5.1 cells. Briefly, a lentiviral vector expressing CD300lf was made by co-transfecting pLVSIN-mCD300lf-IRES-hyg, which is a derivative of pLVSIN-mCD300lf-IRES-puro (31), with a hygromycin resistance gene replacing that of puromycin, and three plasmid constructs, pMDLg/pRRE HIV-1, pMD2G env, and pRSV-Rev (Addgene), into 293T cells. This lentiviral vector was used to transduce the CD300lf expression cassette into Huh7.5.1 cells. The resulting HuhCD300lf cells were selected with 0.2 mg/mL hygromycin, and each single clone isolated by cell sorter (FACSMelody, BD) was tested for its ability to support MNV propagation. Meanwhile, a lentiviral vector expressing MNV VP2 was created by transfecting the constructs, pMDLg/pRRE HIV-1, pMD2G env, pRSV-Rev, and pLVSIN-MNV VP2-IRES-puro into 293T cells. HuhCD300lf clone12-2, which was the best in supporting MNV infection and replication, was used to generate HuhCD300lf VP2 by infection with this MNV VP2-expressing lentiviral vector. The lentiviral vector with the HuhCD300lf clone12-2 was further selected with 5 µg/mL puromycin for cells transduced with MNV VP2 and puromycin expression cassettes.

### Determination of infectivity

The infectivity of wild-type MNV produced from RAW264.7 or HuhCD300lf cells was determined by an assay to determine the 50% tissue-culture infectious dose (TCID_50_). RAW264.7 cells were seeded into 96-well plates (approximately 1 × 10^4^ cells/well) and inoculated with a fivefold pre-diluted virus. After incubation at 37°C with 5% CO_2_ for 4–5 days, infectivity was analyzed by virus-induced cytopathic effect. Virus titers were determined using the Spearman-Karber method.

### Production of recombinant MNV by DNA transfection

Plasmid constructs expressing MNV genes were transfected into 293T cells to generate recombinant viruses. Briefly, 250 ng of each pMNV_S7F_ derivative harboring mutations or deletions in ORF3 region and an equal amount of either pORF3 plasmid were mixed with 1 µL of P3000 solution (Thermo Fisher Scientific) in 50 µL of Opti-MEM (Thermo Fisher Scientific) and then combined with 50 µL of Opti-MEM supplemented with 1.5 µL of Lipofectamine 3000, and incubated for 20 min at room temperature. Before the addition of the lipofectamine-DNA complex, approximately 42% of the culture medium, 0.5 mL in 1.2 mL, of the 293T cells grown in 12-well plates was changed to fresh medium containing 10 mM, adjusted to pH 7.5. After transfection, cells were incubated for 48 hours, and the cell debris in the culture medium was removed by centrifugation at 12,000 × *g* for 10 min at 4°C, and further cleared by filtration through a 0.2 μ filter, to be used as the source of the recombinant virus.

### Detection of the infection events by the mutant viruses

RAW264.7 or RAWVP2 cells infected with either wild-type or mutant MNV were fixed at 48 hpi with 4% paraformaldehyde and suspended in PBS with 0.1% Triton-X. After blocking with 1% BSA/PBS, VPg, and VP1 expression was detected by immunostaining using guinea pig anti-VPg antiserum and rabbit anti-VP1 antiserum, followed by Alexa488-conjugated anti-guinea pig IgG and Alexa594-conjugated anti-rabbit IgG secondary antibodies (Thermo Fisher Scientific), respectively.

HuhCD300lf and HuhCD300VP2 cells were fixed similarly at 48 hpi with 4% paraformaldehyde and suspended in PBS containing 0.1% Triton-X. Samples were blocked with 1% BSA/PBS, and the NS1/2 protein expression was observed by immunostaining with anti-NS1/2 protein antiserum using an epifluorescence microscope (BZ-X 800; KEYENCE). An image of the entire well was taken by concatenating nine images of each well, and positive cells were counted using the built-in image processing software (BZ-X 800 Analyzer; KEYENCE). The percentage of infected cells was calculated by dividing the number of cells expressing the NS1/2 protein by the total number of Hoechst33342-positive cells. Products from each construct at each passage were evaluated in five independent wells.

HuhCD300VP2 cells expressing fluorescent proteins (UnaG or Venus) upon infection with reporter viruses were detected using live-cell imaging with an epifluorescence microscope. Furthermore, Venus proteins were detected simultaneously with viral proteins after cell fixation. The DNA was stained with Hoechest33342. NanoLuc chemiluminescence, carried by the ∆NM-Nluc virus recovered at 72 hpi, was detected according to the manufacturer’s instructions (Nano-Glo Luciferase Assay System, Promega) using a luminometer (EnSight Multimode Plate Reader, PerkinElmer).

Cell viability was determined using a CellTiter96 MTT assay kit (Promega). HuhCD300VP2 was incubated with the supernatant collected at each passage for 48 hours, and the extent of formazan product conversion from MTT by the cellular enzymes was analyzed by measuring the OD_490_ value.

### Confirmation of viral component proteins

Purified wild-type MNV in 2× Sample Buffer (0.125 M Tris-HCl, pH 6.8, 4% SDS, 10% sucrose, 0.01% bromophenol blue, 10% ß-mercaptoethanol) was used as the starting material for separation on 4%–20% Mini-PROTEAN TGX Stain-Free Protein Gels (Bio-Rad). The purified virus was loaded into the sample well and run on the gel in a Mini-PROTEAN Tetra Vertical Electrophoresis Cell for 30 min at 200 V, 50 mA. The gel was imaged using the stain-free application on the ChemiDoc MP (Bio-rad) imager.

### Detection of viral RNA present in the cell supernatant by nested RT-PCR

The wild-type or mutant MNVs in the supernatant of 293T or RAWVP2 cells were immunoprecipitated by rabbit anti-MNV VLP antibody and Ab-Chapcher Mag (ProteNova). The precipitated MNV particles were eluted with 0.1 M glycine-HCl (pH 2.8) and immediately neutralized with 1 M Tris-HCl (pH 8.0). The eluate was purified for viral RNA using a QIAamp Viral RNA Mini (QIAGEN) and treated with Baseline-ZERO DNase (Epicenter) to digest potentially contaminating plasmid DNA. The viral RNA was reverse transcribed by SuperScript III First-Strand Synthesis System for RT-PCR (Thermo Fisher Scientific) with Tx30SXN primer, 5′-GACTAGTTCTAGATCGCGAGCGGCCGCCCTTTTTTTTTTTTTTTTTTTTTTTTTTTTTT-3′. For the 1st amplification reaction, Tx30SXN and MNV-F1, 5′-GCCATGCATGGTGAAAAG-3′ were used as the primers, and MNV-S, 5′-CCGCAGGAACGCTCAGCAG-3′ and MNV-R2, 5′-CAACCACCTTGCCAGCAG-3′ were used for nested PCR in ORF1/2 boundary region to detect MNV RNA. The PCR products were separated on a 1.5% agarose gel, and the bands were visualized using ethidium bromide. The relative intensities of the bands were analyzed using ImageJ software and subsequently a standard curve was calculated from the relative intensities. RNA copy number was determined from the standard curve.

To confirm that the deletions of the ORF3 regions were maintained during the passage of the mutants, virions in the supernatant were similarly immunoprecipitated, and viral RNA was detected using the following set of primers for the nested PCR in the whole ORF3 region: MNV6401-6420 FW, 5′-TCGACTGTGCCCTTCCACAG-3′, and MNV7330-7308 RV, and 5′-TCACAAAAGGTTTCTCTTCCAAC-3′.

### Antisera to MNV

Each polyclonal rabbit antiserum to MNV-S7 N-term, NTPase, VPg, RdRp, and VP1 was produced by *Escherichia coli*-expressed and purified N-term, NTPase, VPg, RdRp, and VP1 immunizations into rabbit and guinea pig, respectively. Anti-MNV-S7 VP2 antiserum was produced with a truncated form of VP2 expressed in *E. coli* that removed the N- and C-terminal disorder regions. The truncated form of VP2 was purified and immunized into rabbit and guinea pig, respectively. Anti-MNV-S7 VLP antiserum was produced by recombinant baculovirus-expressed and purified MNV-S7 VLPs by CsCl sedimentation and immunized into rabbit and guinea pigs.

### Flow cytometric analysis

The expression of NS1/2 and Venus was analyzed using FACS. Cells were detached from the plate with trypsin (Thermo Fisher Scientific). The cells were fixed with 4% paraformaldehyde. Then, 90% methanol was used for permeabilization. The anti-NS1/2 sera were incubated with the cells for 30 min on ice. The cells were then washed and incubated with Alexa Fluor 647 donkey anti-rabbit IgG (Thermo Fisher Scientific) for 30 min on ice. The antisera or secondary antibody was diluted, and the washing steps were carried out in ice-cold PBS without magnesium or calcium salts (PBS (-)). The cells were analyzed using a FACSMelody cell sorter (BD Biosciences) and FlowJo software (BD Biosciences).

### Neutralization assay

The reporter virus (2 × 10^8^ RNA copies) was treated with 100 times diluted rabbit anti-MNV-S7 VLP sera or pre-immune sera for 1 hour at 37°C. The virus-antisera mixture solution was inoculated into HuhCD300lf cells and incubated for 24 hours. After incubation, ∆NM-Venus virus infected cells were analyzed by FACS, and ∆NM-Nluc virus-infected cells were analyzed for their luciferase activity.

### Next-generation sequencing

Genomic viral RNA was purified from culture supernatant including the virus by High Pure Viral RNA Kit (Roche). Purified RNA was used for making the library for sequencing with the NEBNext Ultra RNA Library Prep Kit for Illumina (New England Biolabs) according to the manufacturer’s protocol. Nucleotide sequencing was performed with an iSeq100 sequencer (Illumina) using iSeq100 i1 Reagent Cartridge v2 (Illumina) to generate 151 bp paired-end reads. The FASTQ files were analyzed by using CLC Genomic Workbench 21 (Qiagen-CLC Bio).

### Western blotting

Cell lysates were prepared by adding Laemmli’s SDS/PAGE sample buffer after washing the cells with PBS(−) solution. The lysates were separated on 5%–20% polyacrylamide gels (ePAGEL; ATTO) and transferred onto a PVDF membrane using Trans blot turbo (Bio-Rad). Anti-NS1/2 sera were used as primary antibodies. The anti-NS1/2 sera were labeled with Dylight 650 using LYNX RAPID PLUS DyLight 650 Antibody Conjugation Kit (Bio-rad). For actin detection, anti-actin hFAB rhodamine (Bio-Rad) was used. The ChemiDoc touch (Bio-Rad) was used to detect proteins visualized by Dylight 650 or rhodamine.

## SUPPLEMENTAL MATERIAL

Supplemental material jvi.01261-23-s0001.docx Figures S1 to S6.

ASM does not own the copyrights to Supplemental Material that may be linked to, or accessed through, an article. The authors have granted ASM a non-exclusive, world-wide license to publish the Supplemental Material files. Please contact the corresponding author directly for reuse.
