# Supplementary material for: Production of infectious reporter murine norovirus by VP2 trans-complementation
Source: J Virol. 2024 Jan 16;98(2):e01261-23. doi: 10.1128/jvi.01261-23 (PMC10878090; doi:10.1128/jvi.01261-23)

**Supplemental information**

**Supplemental Fig. 1.** **Characterization of Huh7.5.1, HuhCD300lf and HuhCD300VP2 cells.**

(A)Detached cells were treated with anti-mouse CD300lf antibody and, subsequently with Alexa 647 anti-rabbit IgG and analyzed by flow cytometry. (**B**) The infectivity of wild-type MNV produced from RAW264.7 or Huh7.5.1/CD300lf cells was determined by an assay for the 50% tissue culture infectious dose (TCID_50_). Each cell line was infected with MNV (moi 0.1) and incubated for 96 hours. The infectivity of the virus in each supernatant was determined by TCID_50_ using RAW264.7 cells. Each data bar represents the mean for six independent wells. Error bars denote geometric standard deviation (SD). This experiment was performed one time with six technical replicates. (**C**) VP2 protein detection in purified MNV and HuhCD300VP2 cells was analyzed by western blotting using anti-MNV-S7 VP2 sera. HRP-conjugated anti-rabbit IgG (7074, Cell Signaling Technology) were used as secondary antibodies. The ChemiDoc touch (Bio-Rad) was used to detect protein visualized by Chemi-Lumi One (Nacalai).

**
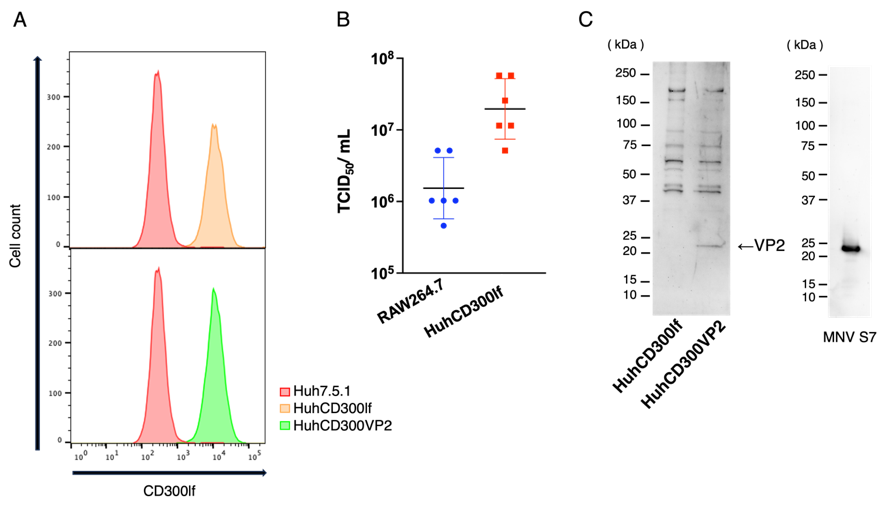
**

**Supplemental Sig. 2 Propagation of MNV variants produced with constructs expressing viral molecular clones harboring defective ORF3 in HuhCD300VP2 cells**

293T cells were co-transfected pORF3 with pMNV_S7F,_ ORF3_stop,_ ORF3∆, ΔN, ΔN_stop_, ΔM, ΔM_stop_, ΔC or ΔC_stop_ and each culture supernatant was transferred to HuhCD300VP2 cells (P+1). Infectious events were detected by immunostaining MNV NS1/2 protein (red) in five independent wells for each construct. As shown in Fig. 4A, the culture medium was further transferred to other HuhCD300VP2 cells (P+2) and to HuhCD300lf cells (P+3 and +4). Images of each well were formed by concatenating nine pictures. The total cells of each well were estimated by counting Hoechst 33342 staining. Representative images before concatenating were shown in Fig. 4B, and the percentages of positive cells were graphed in Fig. 4D. Scale bars are 500 μm.

**
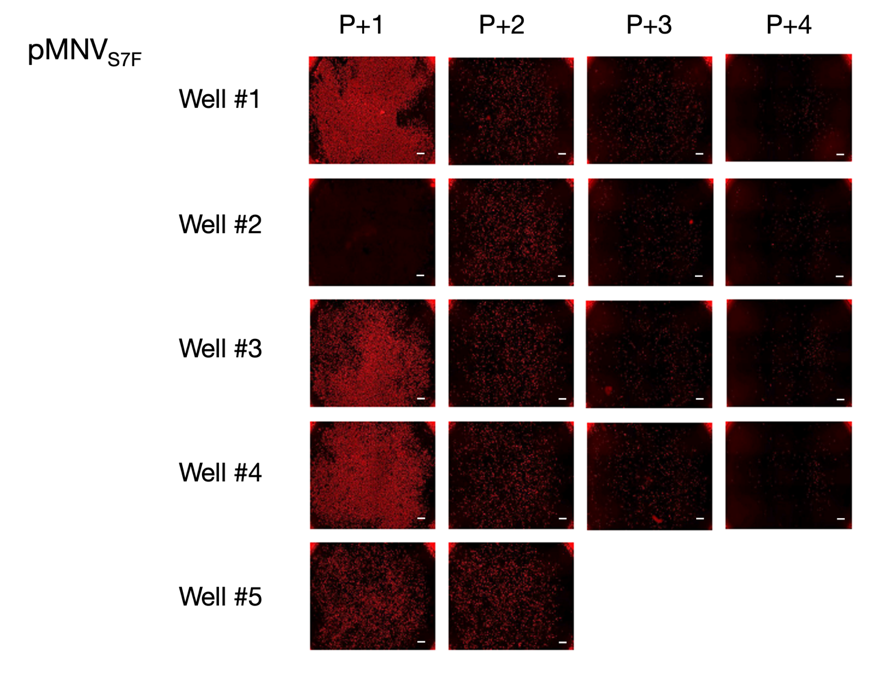
**

**
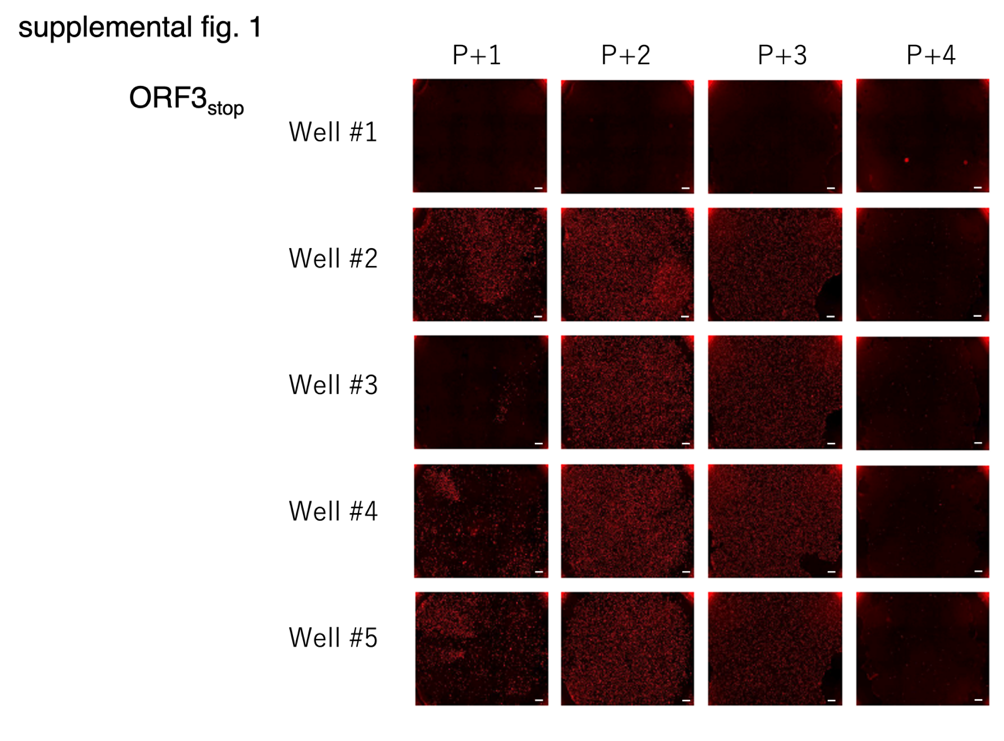
**

**
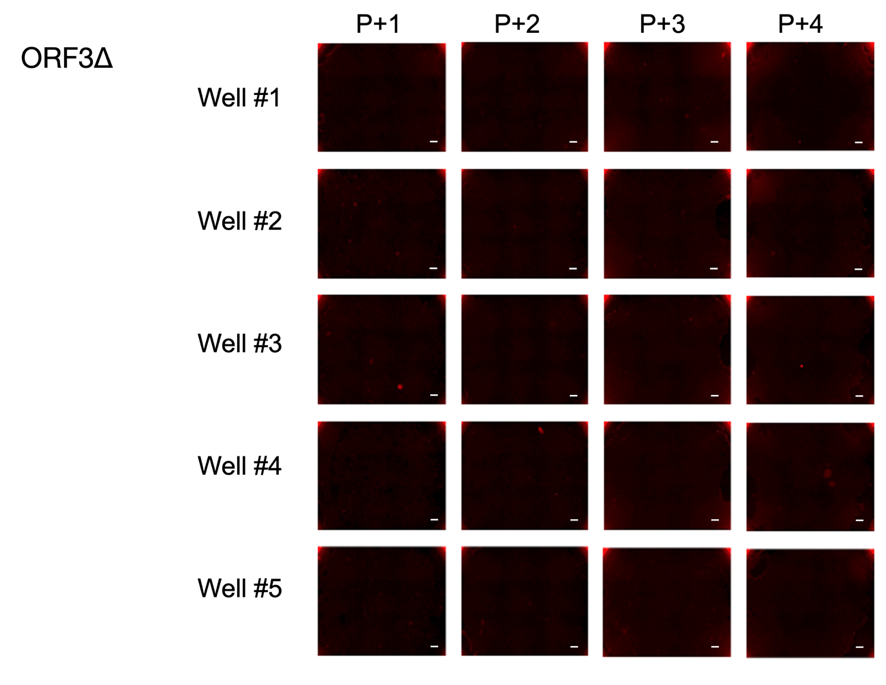
**

**
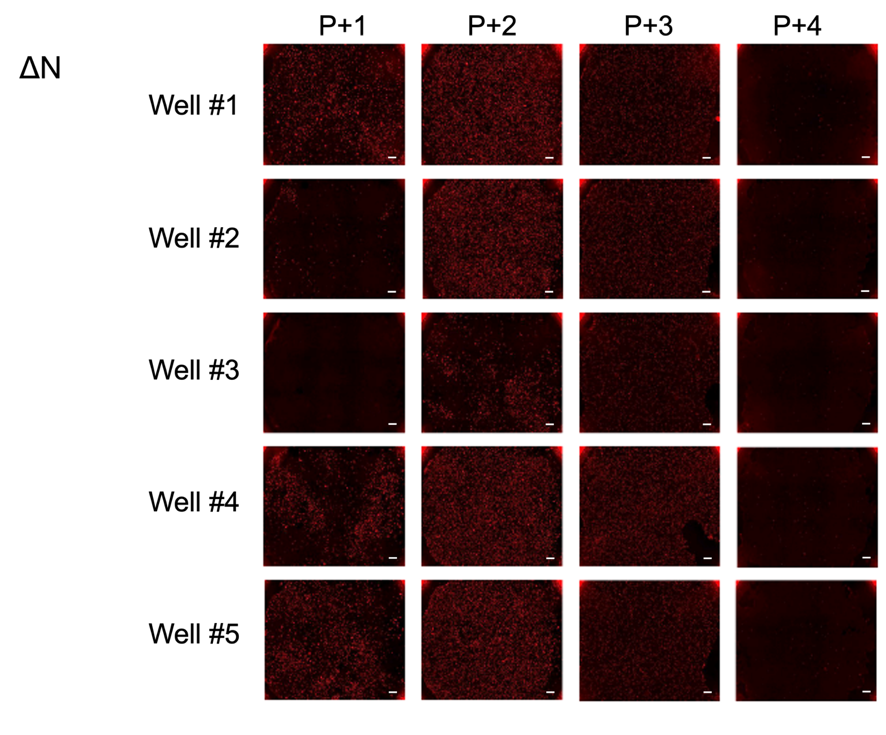

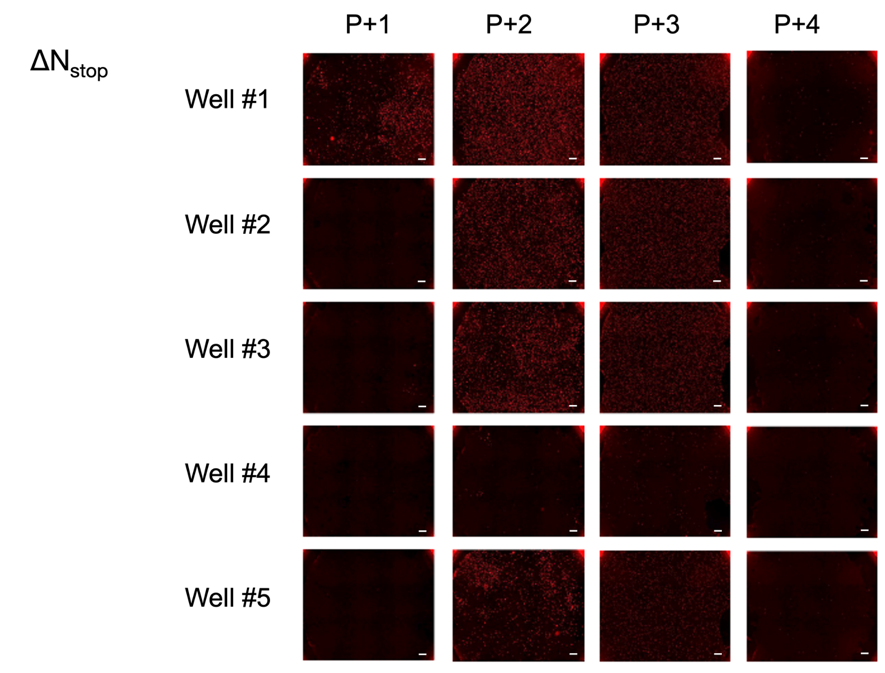
** **
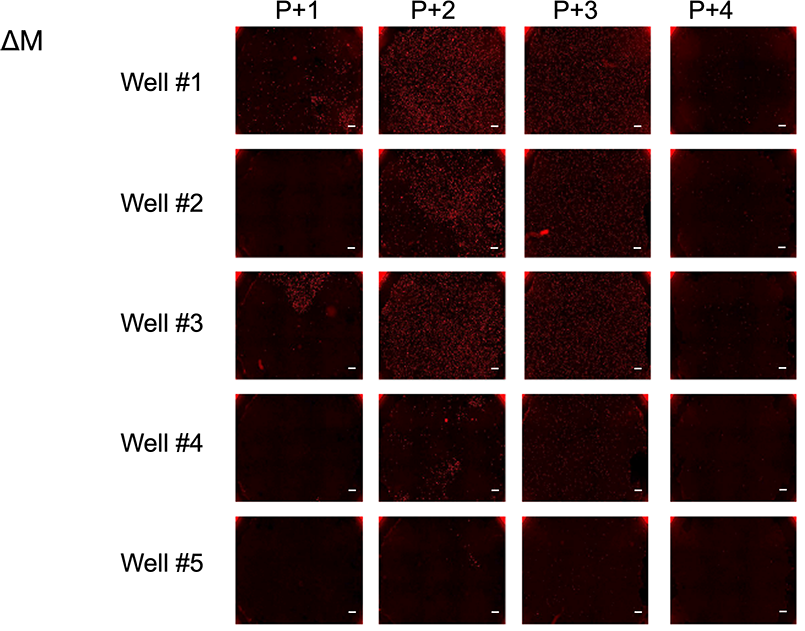

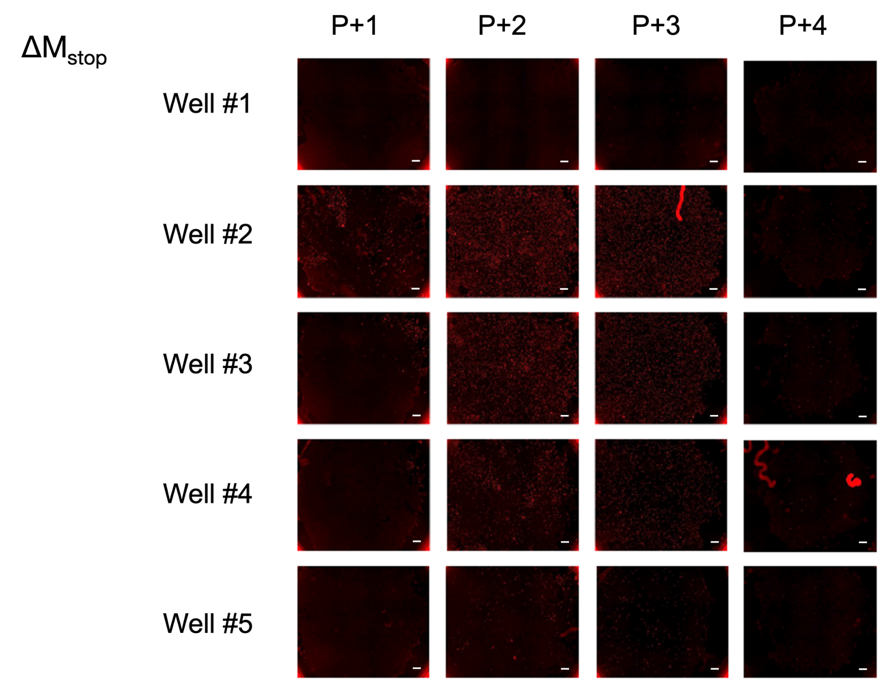

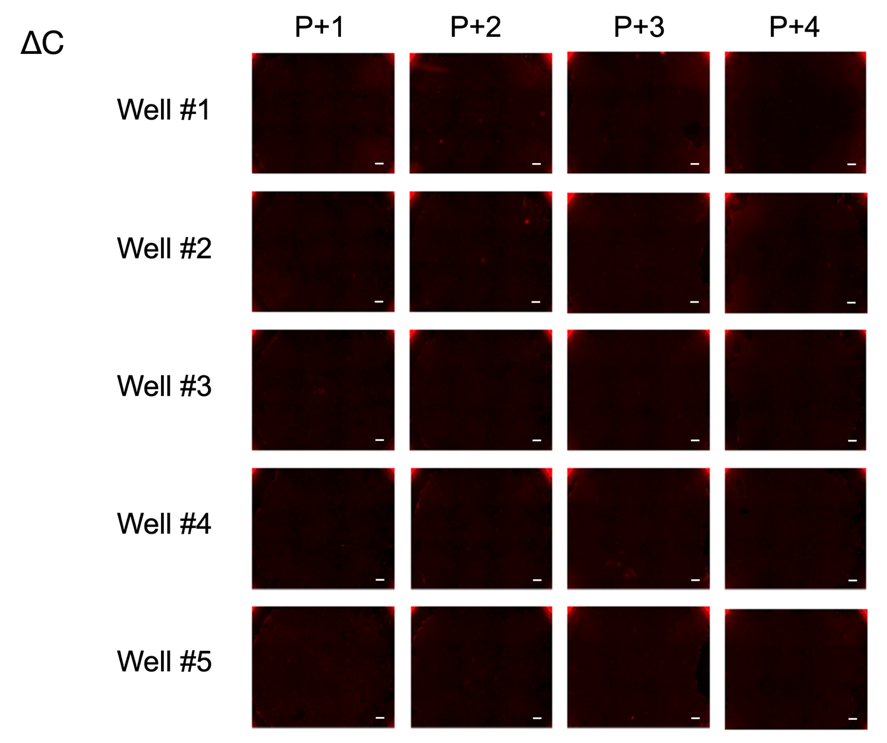

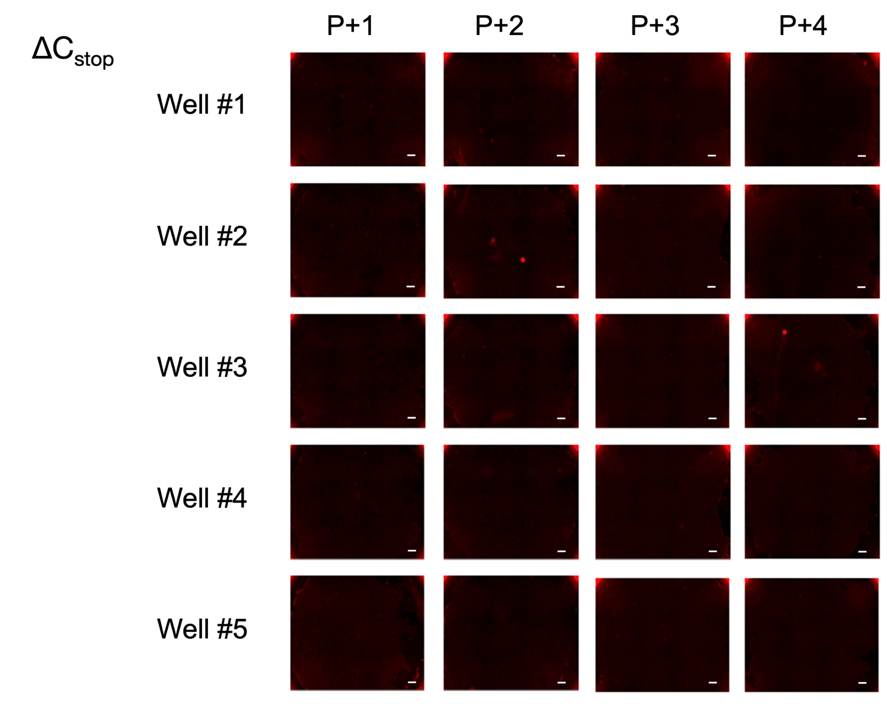

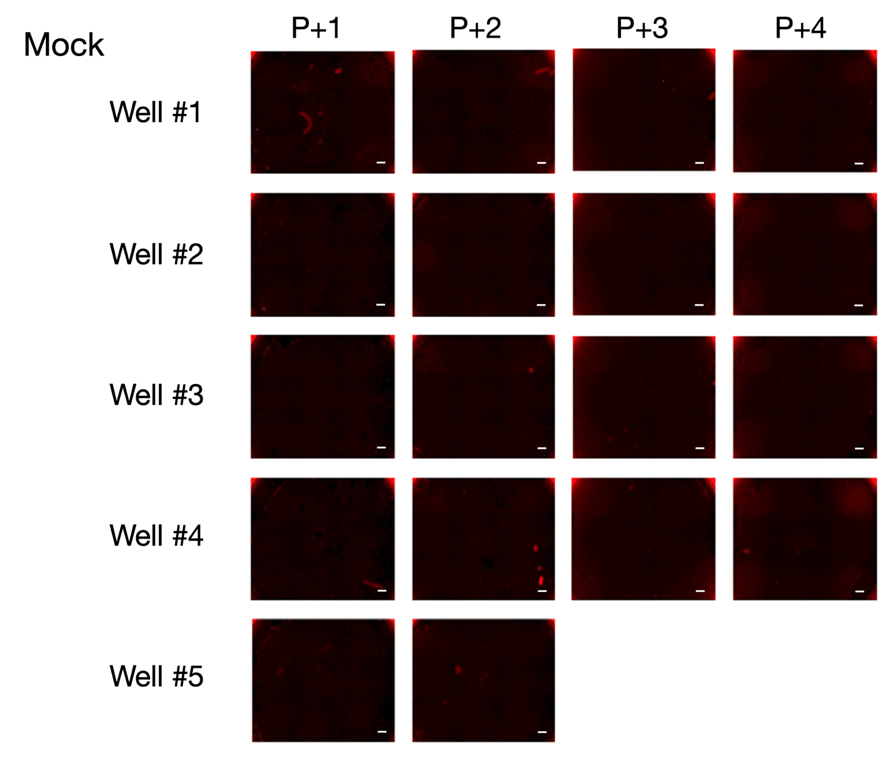
**

**Supplementary Fig. 3. Whole-genome sequence of propagated MNV_stop_ (A), ∆NM-Venus virus (B), ∆NM-UnaG virus (C) and ∆NM-Nluc virus(D).**

Next generation sequencing (NGS) was performed for sequencing of whole genome of each virus, and the reads from each virus were mapped on each reference sequence with CLC genomics workbench ver. 21. The arrows indicated nucleotides changes observed in ORF2 region.

**(A)**

**
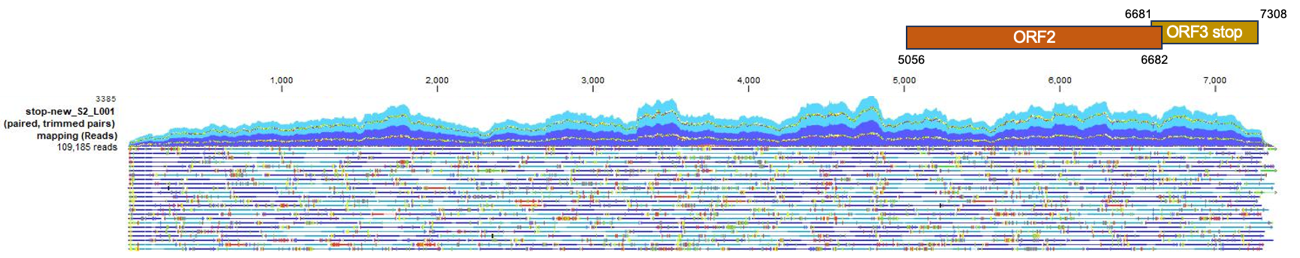
**

**(B)**

**
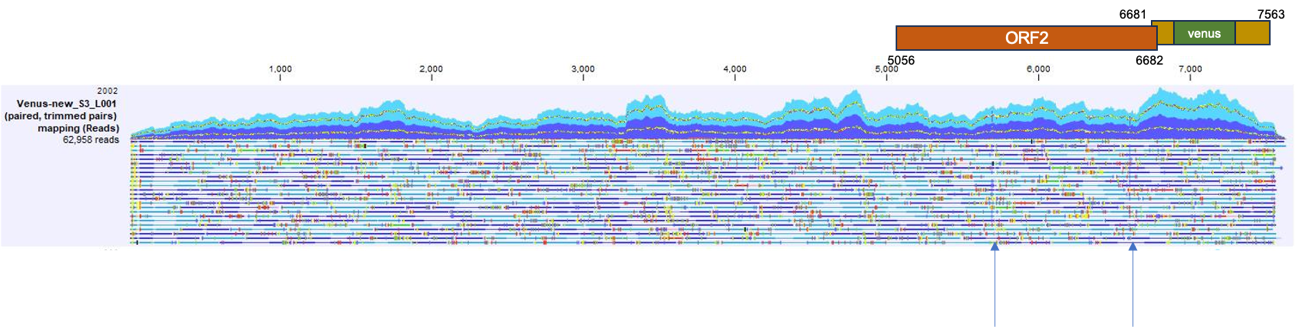
**

**(C)**

**(D)**

**
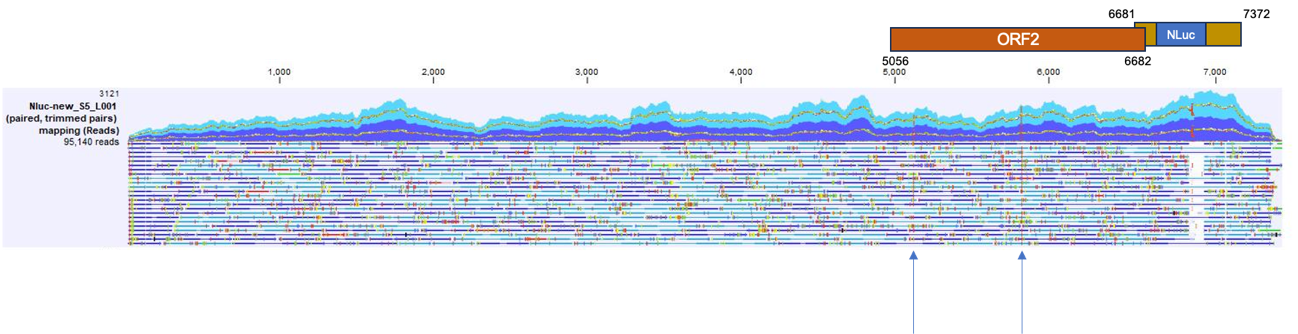
Supplementary Fig. 4. The cells expressing NS1/2 protein or Venus protein were counted by IF from images the BZX software or flow cytometry**

(**A**) Cell counting of NS1/2 protein expressing cells and Venus expressing cells by BZX software. ∆NM-Venus virus infected HuhCD300lf cells were stained with anti NS1/2 protein antibody and subsequence fluorescent secondary antibody. The software counts nuclei stained with Hoechst34432 as total cells in the extended image, because nuclei signal was too small to be counted in same scale images for counting NS1/2- and Venus-positive cells. Yellow dots indicated the detected cells by each signal with the software. The extension image on right side indicated that the software missed some signals from Venus-expressing cells. (**B**) After immunostaining against the NS1/2 protein, flow cytometry was applied to count the cells expressing each protein. Each experiment was performed with two technical replicates. Data from a representative experiment are shown. (**C**) The average ratio of two technical replicates measured by each method.

**Supplementary Fig. 5. Population of Venus expression of different passages of ∆NM-Venus virus in HuhCD300VP2 cells.**

∆NM-Venus virus (1×10^9^ copies) in different passages (P+6, P+7, and P+8) was infected into HuhCD300VP2 cells (1×10^6^ cells), and cells were collected at 24 and 48 hpi. Each data bar represents the mean of three independent wells. Error bars denote SD. This experiment was performed one time with three technical replicates.

**Supplementary Fig. 6. Transition of the population of NS1/2 expression and Venus expression in HuhCD300lf cells.**

∆NM-Venus virus (1×10^8^ copies) were infected into HuhCD300lf cells (1×10^5^ cells), and cells were collected every hour from 6 to 14 hpi.


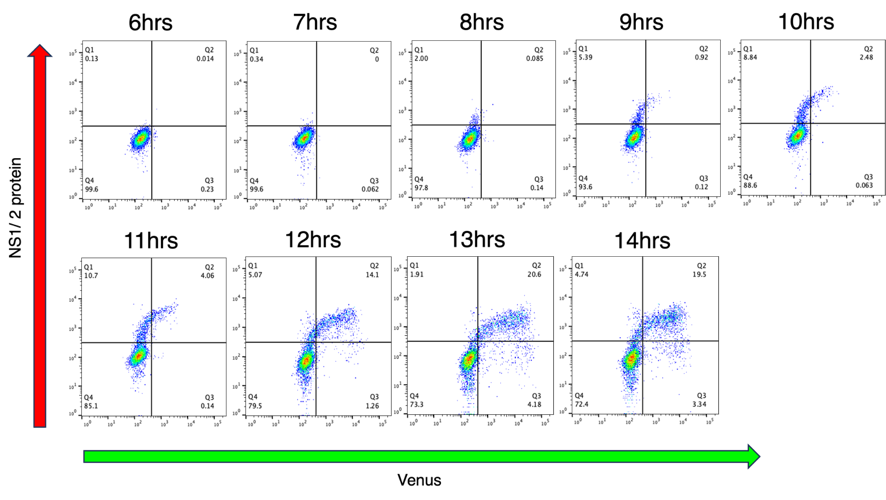

Supplement: Supplemental material — Figures S1 to S6. [file jvi.01261-23-s0001.docx]
